# Supplementary material for: Caesarean birth in public maternities in Argentina: a formative research study on the views of obstetricians, midwives and trainees
Source: BMJ Open. 2022 Jan 25;12(1):e053419. doi: 10.1136/bmjopen-2021-053419 (PMC8796244; doi:10.1136/bmjopen-2021-053419)
Supplement: Author's manuscript [file bmjopen-2021-053419.draft_revisions.pdf]

BMJ Open

BMJ Open is committed to open peer review. As part of this commitment we make the peer review history of every article we publish publicly available.

When an article is published we post the peer reviewers' comments and the authors' responses online. We also post the versions of the paper that were used during peer review. These are the versions that the peer review comments apply to.

The versions of the paper that follow are the versions that were submitted during the peer review process. They are not the versions of record or the final published versions. They should not be cited or distributed as the published version of this manuscript.

BMJ Open is an open access journal and the full, final, typeset and author-corrected version of record of the manuscript is available on our site with no access controls, subscription charges or pay-per-view fees (http://bmjopen.bmj.com).

If you have any questions on BMJ Open's open peer review process please email info.bmjopen@bmj.com

BMJ Open

Caesarean birth in public maternities in Argentina: a formative research study on the views of obstetricians, midwives, and trainees.

Journal:	*BMJ Open*
Manuscript ID	bmjopen-2021-053419
Article Type:	Original research
Date Submitted by the Author:	13-May-2021
Complete List of Authors:	Perrotta, Carla; University College Dublin, School of Public Health, Physiotherapy and Sport Sciences<br>Romero, Mariana; CONICET (Consejo Nacional de Investigaciones Científicas y Tecnológicas)<br>Sguassero, Yanina; Centro Rosarino de Estudios Perinatales<br>Straw, Cecilia; CONICET (Consejo Nacional de Investigaciones Científicas y Tecnológicas)<br>Righetti, Natalia; CEDES, Health, Economy and Society<br>Gialdini, Celina; Centro Rosarino de Estudios Perinatales<br>Betran, Ana Pilar; World Health Organization, Department of Reproductive Health and Research<br>Ramos, Silvina; CEDES, Health, Economy and Society
Keywords:	Maternal medicine < OBSTETRICS, PUBLIC HEALTH, Organisation of health services < HEALTH SERVICES ADMINISTRATION & MANAGEMENT

SCHOLARONE™
Manuscripts

I, the Submitting Author has the right to grant and does grant on behalf of all authors of the Work (as defined in the below author licence), an exclusive licence and/or a non-exclusive licence for contributions from authors who are: i) UK Crown employees; ii) where BMJ has agreed a CC-BY licence shall apply, and/or iii) in accordance with the terms applicable for US Federal Government officers or employees acting as part of their official duties; on a worldwide, perpetual, irrevocable, royalty-free basis to BMJ Publishing Group Ltd ("BMJ") its licensees and where the relevant Journal is co-owned by BMJ to the co-owners of the Journal, to publish the Work in this journal and any other BMJ products and to exploit all rights, as set out in our licence.

The Submitting Author accepts and understands that any supply made under these terms is made by BMJ to the Submitting Author unless you are acting as an employee on behalf of your employer or a postgraduate student of an affiliated institution which is paying any applicable article publishing charge ("APC") for Open Access articles. Where the Submitting Author wishes to make the Work available on an Open Access basis (and intends to pay the relevant APC), the terms of reuse of such Open Access shall be governed by a Creative Commons licence – details of these licences and which Creative Commons licence will apply to this Work are set out in our licence referred to above.

Other than as permitted in any relevant BMJ Author's Self Archiving Policies, I confirm this Work has not been accepted for publication elsewhere, is not being considered for publication elsewhere and does not duplicate material already published. I confirm all authors consent to publication of this Work and authorise the granting of this licence.

For peer review only

Caesarean birth in public maternities in Argentina: a formative research study on the views of obstetricians, midwives, and trainees.

Perrotta Carla, Romero Mariana, Sguassero Yanina, Straw Cecilia, Gialdini Celina, Righetti Natalia, Betrán Ana- Pilar, Ramos Silvina

Carla Perrotta, PhD. Corresponding author. School of Public Health, University College Dublin, National University of Ireland, Dublin, Ireland. Address: Woodview House, Belfield campus. E-mail: carla.perrotta@ucd.ie +353 83 84 99147

Mariana Romero, MD. Centro de Estudios de Estado y Sociedad (CEDES)- CONICET (Consejo Nacional de Investigaciones Científicas y Tecnológicas), Buenos Aires, Argentina. E-mail: mromero@cedes.org

Yanina Sguassero, PhD. Centro Rosarino de Estudios Perinatales, Rosario, Argentina. E-mail: ysguassero@crep.org.ar

Cecilia Straw, PhD. Facultad de Ciencias Sociales, Universidad de Buenos Aires. Centro de Estudios de Estado y Sociedad (CEDES)- CONICET (Consejo Nacional de Investigaciones Científicas y Tecnológicas), Buenos Aires, Argentina. E-mail: Cecilia.straw@gmail.com

Celina Gialdini, MD. Centro Rosarino de Estudios Perinatales, Rosario, Argentina. E-mail: cgialdini@crep.org.ar.

Natalia Righetti, MSc. Centro de Estudios de Estado y Sociedad (CEDES), Buenos Aires, Argentina. E-mail: natirighetti@gmail.com

Ana Pilar Beltrán, MD. UNDP, UNFPA, UNICEF, WHO, World Bank Special Program of Research, Development and Research Training in Human Reproduction, Department of Reproductive Health and Research, World Health Organization, Geneva, Switzerland. E-mail: betrana@who.int

Silvina Ramos, MSc. Centro de Estudios de Estado y Sociedad (CEDES), Buenos Aires, Argentina. E-mail: silvinaramosarcoiris@gmail.com

For peer review only
For peer review only - http://bmjopen.bmj.com/site/about/guidelines.xhtml

Abstract

Objectives To explore obstetricians, midwives, and trainees' perceptions of CS determinants in the context of public obstetric care services provision in Argentina. Our hypothesis is that known determinants of CS use may differ in settings with limited access to essential obstetric services.

Setting We conducted a formative research study in nineteen public maternity hospitals in Argentina. An institutional survey assessed the availability of essential obstetric services. Subsequently, we conducted online surveys and semi-structured interviews to assess the opinions of providers on known CS determinants.

Results

Obstetric services showed an adequate provision of emergency obstetric care but limited services to support women during birth. Midwives, with some exceptions, are not involved during labour. We received 680 surveys from obstetricians, residents, and midwives (response rate of 63%) and interviewed 26 key informants. Six out of ten providers (411, 61%) indicated that the use of CS is associated with "*the complexities of our caseload*". Limited pain management access was deemed a potential contributing factor for CS in adolescents and first-time mothers. Providers have conflicting views on the adequacy of training to deal with complex or prolonged labour Obstetricians with more than ten years of clinical experience indicated that fear of litigation was also associated with CS. Overall, there is consensus on the need to implement interventions to reduce unnecessary CS.

Conclusions

Public Maternity Hospitals in Argentina have made significant improvements in the provision of emergency services. The environment of service provision does not seem to facilitate the physiological process of vaginal birth. Providers acknowledged some of those challenges.

Funding

UNDP-UNFPA-UNICEF-WHO-Special Funding for research. University College Dublin Seed Funding.

Trial registration IS002316

For peer review only - http://bmjopen.bmj.com/site/about/guidelines.xhtml

Article Summary

Strengths and limitations of this study

- This study is one of the few studies exploring the perceptions of providers working in low-resourced settings in middle-income countries on the determinants of increasing trends of CS.
- Large sample and representation of all professionals and obstetric tasks working in low-resourced settings.
- Cluster analysis allowed to describe the response variability across professional groups in relation to specific CS determinants.
- The use of Formative Research is a valuable tool to inform the design and implementation of future interventions.
- Even though the response rate is good (63%) those who did not respond may have different views on the determinants of CS.
- Some participant hospitals had lower response rate.

For peer review only

Background

In recent decades, there has been a sustained and unprecedented increase in caesarean section (CS) rates worldwide, in particular −but not exclusively− in the Latin America region.[1] Argentina, a middle-income country, reported rates between 27% and 52% within the public sector in 2017.[2] Similarly, Brazil registered national rates of 55%, with private providers close to 90%.[3] These regional trends cause concern and call for a scaling-up of interventions to reduce unnecessary CS.

The drivers increasing rates of CS are multiple. The most frequently cited determinants stem from financial incentives, the organisation of services, fear of litigation, and professional attitudes towards CS.[4–10] Along with clinical interventions, non-clinical interventions such as antenatal education, continuous emotional support during labour, audit and feedback, and the implementation of clinical guidelines have shown modest but statistically significant reductions in CS rates.[11,12] The World Health Organization (WHO) recommends implementing non-clinical interventions to reduce unnecessary surgical births and it encourages the incorporation of an initial formative research process to tailor interventions to local contexts, ensure implementation feasibility, and anticipate specific barriers or identify facilitators.[13,14]

Argentina is a federal country with a two-tier health system: a public system financed by government funds and a private system mix of social insurance and private insurance. Virtually all births occur at healthcare institutions (99.3%), and 64% of them within the public health system.[2] Specialists lead obstetric services in Obstetrics and Gynaecology (OB/GYN) and the role of midwives depends on how the hospital organises its services and human resources. Midwives provide antenatal care and depending on the institution, can assist or lead low-risk vaginal births.

For peer review only - http://bmjopen.bmj.com/site/about/guidelines.xhtml

We conducted formative research to enrich our understanding of the reasons for the sustained increase in CS use in Argentina's public sector to tailor context-specific interventions.[15] We applied a mixed-methods approach to explore multiple layers and perspectives from both women and health providers, as well as service delivery characteristics. Formative research has its origins in ethnographic studies and uses various data-gathering processes. The rationale behind this approach is to consider the peculiarities of the setting to better tailor suitable interventions and, at the same time, to catalyse the target population into change.[16–18.]

This article will focus on the perspectives of specialists in OB/GYNs, midwives and OB/GYN residents (trainees) on determinants of CS use in Argentina's public maternity hospitals.

Methods

A comprehensive description of the methodology has been published elsewhere.[15] Briefly, we used a research approach that combined quantitative and qualitative data gathering techniques conducted by a multidisciplinary team.

The contextual factors of the study refer to the adequacy of services to provide emergency obstetric care, human resources availability, and women's access to comprehensive obstetric care like antenatal education, companionship during birth, and holistic pain management.

The eligibility criteria for hospitals to participate in the study were: a) public hospitals with ≥1000 annual childbirths, b) caesarean section rate higher than 27% in 2016, and c) use of the National Perinatal Information System.[19,20] Out of the 88 potentially eligible institutions, 24 were non-randomly selected to represent the six Argentinian regions. Nineteen agreed to participate and obtained approval from local Ethics Committees (Figure 1), and five institutions declined to participate. Finally, five Argentinian regions were included in this study: Centre, North, Northeast and Buenos Aires City.

The project was approved by the Independent Ethics Committee of Centro Rosarino de Estudios Perinatales and by the provincial Ethics Committees and/or the Teaching and Research Committees at each of the selected hospitals pursuant to the requirements in each jurisdiction. It was also approved by two Ethics Committees of WHO, the Research Project Review Panel of the UNDP/UNFPA/UNICEF/WHO/World Bank Special Programme of Research, Development and Research Training in Human Reproduction (HRP) at the Department of Sexual and Reproductive Health and Research of WHO, and the WHO Research Ethics Review Committee, Geneva, Switzerland. In Argentina, the research protocol was registered in the RENIS database (number IS002316).

For peer review only - http://bmjopen.bmj.com/site/about/guidelines.xhtml

The report of this research follows the Standards for Reporting Qualitative Research and Formative Research.[21,22.]

Subjects and data collection

Multiple data sources were used: an institutional survey, a health care provider online survey, and semi-structured interviews with key informants. The head of OB/GYN identified a spokesperson who led the fieldwork and introduced the project to health team members at each participating hospital.

Institutional survey

The selected champion in each hospital completed the institutional survey. The survey gathered information on the number of live births in 2017, the number and type of providers involved in maternal care, hospital CS rates, institutional infrastructures such as the number of beds, availability of services to safely manage obstetric emergencies, availability of antenatal education for women, and pain management during labour and birth.

Online survey for healthcare providers

Eligible participants for the online survey included all specialised physicians, obstetricians, and residents at Obstetrics & Gynaecology services at participating hospitals. Both permanent staff and shift personnel were included.

The health professionals' online survey included participation consent (mandatory answer) and 32 questions: 26 with pre-established options and six open-ended questions. The survey gathered the following data: role and healthcare work, including the number of deliveries/caesarean sections performed in a week; perception of determining factors for the use of caesarean section related to the institution, health professionals and pregnant women; frequency of use of monitoring cycles; improvement in quality of care; and Robson Classification knowledge, among others.[23.]

The piloting process involved ten hospitals and asked participants to indicate if they would like to add anything. Fifteen providers suggested that inadequate pain management during labour is a contributing factor; thus, we incorporated it in the list of determinants of CS use ("*lack of pain management interventions is associated with the use of CS*").

We sent an email with the link to the survey to each eligible subject who provided an email address; the text included a brief presentation of the study with a consent form. The respondents' gender or sex was not recorded to ensure anonymity. There were three weekly reminders. Fieldwork was carried out from July 2018 to August 2019.

Semi-structured interviews for key informants

Out of the 19 hospitals participating in the study, we drew a convenience sample of five hospitals representing the different regions. In those hospitals, we conducted in-depth interviews after the on-

For peer review only - http://bmjopen.bmj.com/site/about/guidelines.xhtml

line survey with key informants, including the heads of departments or services, specialists in OB/GYN, midwives, and residents. The interviewers were researchers with experience in qualitative methods and in maternal health and organisational culture. The interviews were conducted either in person or by video call and recorded under informed consent.

Data analysis

We described variables using standard statistical parameters. Continuous variables were presented as means and standard deviations (SDs), whereas categorical variables were presented as numbers and percentages. Human resources data was standardised to 1000 live births in 2017.

For the online survey, answers were grouped into three categories: disagreement (strongly disagree and disagree), neutral, and agreement (completely agree and agree), and were described using frequencies. The statements with modest consensus –those with ≤ 50% agreement or disagreement– were analysed using cluster analysis to examine if professional groups or any institutional variable explained the variance. Given the different types of variables assessed, a Gower's distance matrix was calculated. It was visualised using the t-Distributed Stochastic Neighbour Embedding (t-SNE, 1) and clustered with Partition around medoids (PAM) with k = 4. All statistical analyses were performed using R version 3.6.3.[24–26]

We transcribed the recorded in-depth interviews. Transcripts were independently coded by two researchers with experience in qualitative analysis. We undertook an in-depth framework analysis of the qualitative data focusing on determinants of CS and institutional factors.[27] We compared key themes emerging from the interviews with the online survey results, expanding to supplement survey findings when appropriate. Throughout the results section, we report verbatim extracts to validate and illustrate specific issues, provide further explanations, or include themes that were not addressed by the online survey.

Patient and public involvement

The development of the protocol used evidence from qualitative research exploring women experience with obstetric services in public maternity hospitals. Once the study has been published, the results will be disseminated for professional, non-professional audiences in participant provinces as well as to non-governmental organizations overseeing maternal care in the Latin American region.

Results

Women giving birth in the 19 participant hospitals represented 19% of the total live births in Argentina's public sector in 2017 (Figure 1).

Table 1 shows the characteristics of the 19 hospitals in 2017 as reported by the key informants in each institution. The percentage of live births by CS in the study hospitals ranged from 29% to 40% (Table 1). The hospitals had similar services; all of them had laboratory, neonatal intensive care units,

For peer review only - http://bmjopen.bmj.com/site/about/guidelines.xhtml

adequate access to emergency CS, and resources to manage obstetric emergencies, including safe blood transfusion services. All hospitals but one had limited access to pain management during birth, insufficient provision of antenatal education, and restricted companionship during CS. They differed on the number of healthcare providers per 1,000 live births, with a median of seven obstetricians/1000 live births and 2·8 midwives/1000 live births. Two hospitals reported a very low staffing ratio −one hospital reported 1 obstetrician/1000 live births−; and the largest number of obstetricians was 23/1000 live births in one hospital. All the professionals received a fixed monthly salary unrelated to performance.

For peer review only

Figure 1 Study Flowchart

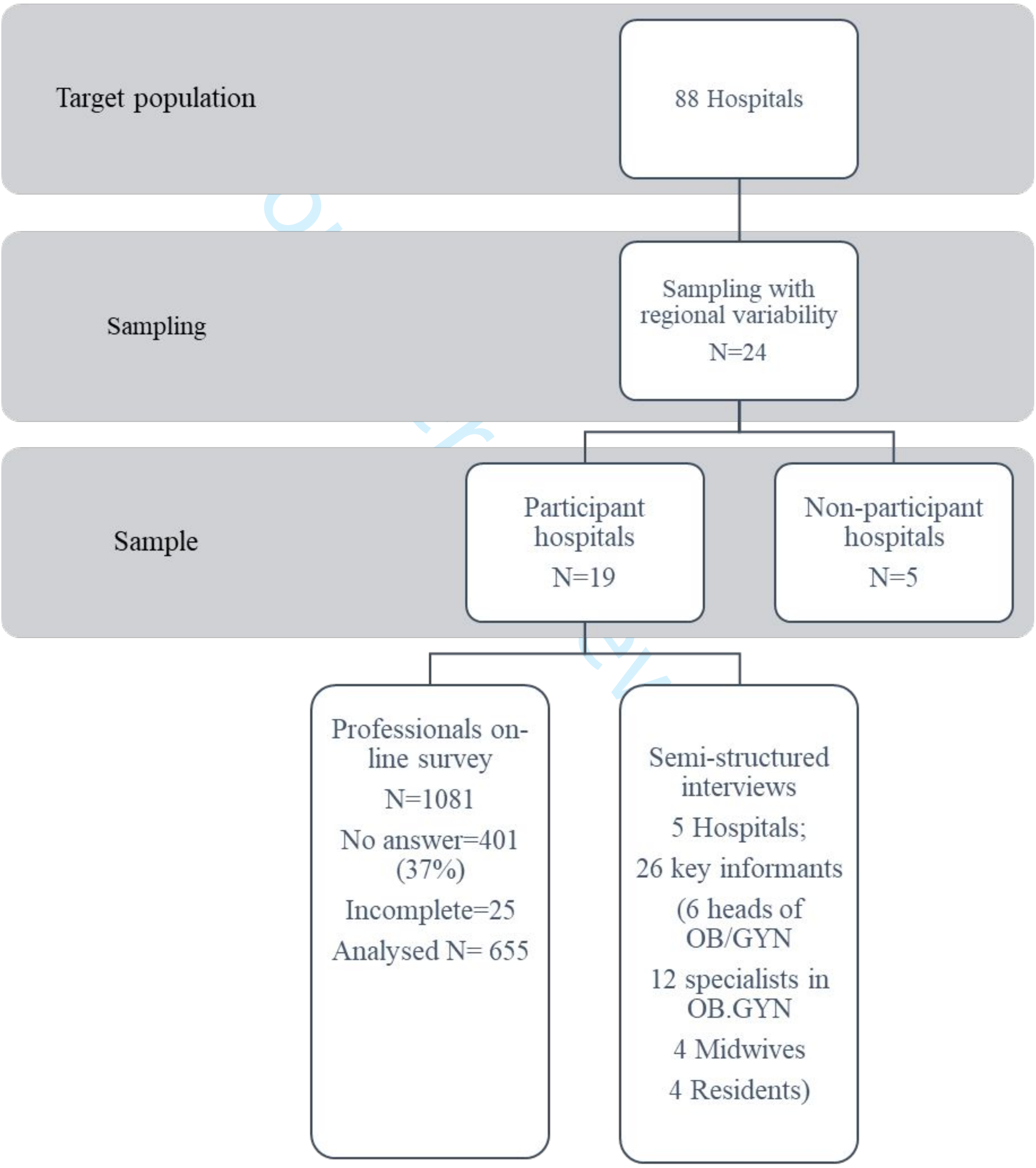

Table 1 Characteristics of participant hospitals (N=19)

**Variables**	**Hospitals**<br>**N (%) Mean (SD) Median (range)**
Type of institution	
Maternity	7 (37%)
General hospital	12 (63%)
Number of beds †	
Obstetrics	44 (1-93)
Neonatal Intensive Care Unit	18 (1-50)
Number of delivery rooms or units of labour, delivery, and recovery†	6 (1-16)
Number of live births	52633<br>2355 (1025-9633) †
Caesarean Sections (CS)	
Number of CS	21102
CS rate†	37·2 (27-50)
Protocol for vaginal delivery after CS	15 (83%)
Residency Program	
OB/GYNs	18 (90%)
Midwives	7 (36%)
Number of professionals†	
OB/GYNs	20 (2-67)
Residents†	13 (0-31)
Midwives†	12 (0-30)
Number of professionals/ 1000 live births†	
OB/GYNs	7 (1-23)
Residents	5·8 (0-15)
Midwives	2·8 (0-18)
Midwives' availability (24/7)	13 (68%)
Midwives participating in low-risk births	9 (45%)
Pain management interventions	
Access to non-opioids	10 (52%)
Access to epidural (24/7)	9 (47·4%)
Access to opioids	12 (63%)
Relaxation techniques	4 (21·1%)
Access to hot shower	9 (47·4%)
Access to massages	3 (15·2%)
Access to companionship (24/7)	
During labour	14 (73%)
During delivery	16 (84%)
During CS	2 (10%)
Availability of prenatal education (morning, afternoon, Monday through Friday)	1 (5·5%)

*Data is shown as N (%) unless otherwise indicated.
†median (range)
OB/GYN: Specialist in Obstetrics and Gynaecology

We received 680 online surveys (response rate of 63%; range 19%-84%). We excluded 25 due to missing data related to professional background, which resulted in 655 completed surveys to analyse (Figure 1).

The completed surveys were from OB/GYN specialists (N=327), midwives (N=161) and residents (N=167). Cluster analysis allowed us to identify four groups based on tasks and years of experience,

For peer review only

For peer review only - http://bmjopen.bmj.com/site/about/guidelines.xhtml

and profession. Group 1 (N=247) included OB/GYNs, who worked for a mean of 11 years and attended 2-10 birth per week (any type). Group 2 had more experienced OB/GYNs (N=80) and midwives (N=23) (mean 22 years of professional experience) involved either in antenatal care, teaching, or management duties. Group 3 consisted of residents (N=167) with three years of experience participating in more than ten deliveries per week. Group 4 included midwives (N=138) with a mean professional practice of 12·5 years in assisting vaginal deliveries (Table 2). We conducted 26 semi-structured interviews in total with six heads of OB/GYN departments, 12 specialists in OB/GYN, four midwives and four residents.

Table 2 Online survey clusters according to clinical experience (years) and main duties

	Median years of professional experience	Main duties	N=655
**Group 1 OB/GYNs**	11	Assistance of all delivery modes. 2-10 deliveries per week	247
**Group 2 OB/GYNs and midwifes**	22	Antenatal care, research, supervisory tasks, education	103
**Group 3 Residents**	3·5	Assistance of all delivery modes. More than 10 deliveries per week	167
**Group 4 Midwifes**	12·5	Assistance of all deliveries 2- 10 deliveries per week	138

OB/GYN: Specialist in Obstetrics and Gynaecology

Six out of ten participants (411, 61%) agreed that the use of CS is associated with "*the complexities of our caseload*" (Table 3). Additionally, key informants point out diagnostic innovations and comorbidities as determinants of CS use.

"Nowadays, we have more diagnostic procedures during pregnancy [...] Maybe, in the past, women had a vaginal birth with worse clinical outcomes [...] yes, the number of CS has increased, but the neonatal outcomes have improved" (Hospital 2, OB/GYN).

"The main factor for CS is clinical. We see more women with severe comorbidities. Also, if they have had a previous CS and comorbidity, the delivery is a CS [...]." (Hospital 3, OB/GYN).

Shortage of skilled professionals was considered a determinant of the overuse of CS by 12.8% of the sample while 16% agreed that insufficient training for complicated births was a determinant.

A similar level of agreement appeared regarding inadequate skills to conduct vaginal birth after CS (VBAC) (N=84, 12.4 %) (Table 3). However, when asked if CS is associated with residents' lack of training in performing complex deliveries, the professional groups differed in their answers: 30% of midwives agreed that there is an association (N=39, 30%) while only 5% of residents agreed (Table 4).

The semi-structured interviews allowed us to explore further whether the number, skills, and experience of obstetricians, midwives, and residents influence the use of CS. Among the interviewees, there was consensus that professionals are well trained; however, midwives perceived themselves as

For peer review only - http://bmjopen.bmj.com/site/about/guidelines.xhtml

less prone to intervene and considered that the medical professionals were trained with a more interventionist mindset.

"We have to fight every day as doctors tend to intervene. Now we have a training program on foetal medicine, and most doctors perform a foetal ultrasound. I notice more and more the tendency to intervene" (Hospital 1, midwife).

The semi-structured interviews also revealed challenges in training future generations of obstetricians to perform instrumental deliveries when needed.

"Instrumental deliveries are tough, and no one wants to do them. Before, it was a common practice; now, people are cautious. In the collective imagination of society, the forceps can kill or harm the baby. Even judges, lawyers have that idea… [And the training to conduct instrumental deliveries?] … little as we don't do them" (Hospital 4, OB/GYN).

"A resident can complete their training -if lucky- performing five forceps. Maybe a handful of obstetricians at the ward have the skills to use forceps. Doctors prefer a CS as it can quickly solve the clinical problem" (Hospital 2, Resident).

In most hospitals, key informants did not support the idea that scarcity of human resources is relevant to the increase of CS. However, in two hospitals with either no midwives on their teams or a very low staff-to-live-birth ratio, clinicians did recognise it as a contributing factor. In the latter two hospitals, busy shifts with only one doctor on-call were described. In those circumstances, if a woman presented with failing-to-progress labour, the decision may switch to a surgical delivery (verbatim not shown).

Questions related to women's preferences and first-time mothers' anxiety due to lack of preparation for birth had the highest number of neutral responses. We found variability between professional groups. 17% of midwives agreed with the statement "*women demand a CS"* compared to 30% of obstetricians and 28% of residents. For the statement "*the use of CS in first-time mothers is related to anxiety*", 38% of the obstetricians, 34.4% of residents and 29% of midwives agreed it could be considered a determinant of CS use (Table 4).

Key informants discussed both matters:

"*We have requests both ways. Patients that want vaginal birth at all cost and patients that want CS. We work under significant pressure. Patients have changed…*" (Hospital 4, OB/GYN).

Lack of adequate access to pain management interventions as a reason for CS did not reach consensus in the on-line survey; 31% agreed, 44% disagreed, and 12.7% had a neutral opinion, with statistically insignificant variation between responses (Table 3). Key informants helped to understand the role of limited access to epidurals and other pain management strategies like hot water, relaxation, or massage.

There was some consensus that the absence of these options might influence women's requests for a CS, especially in adolescent mothers.

For peer review only - http://bmjopen.bmj.com/site/about/guidelines.xhtml

"If a patient is in pain, I won't specifically say 'OK, this patient needs a CS because of the pain'. It does not affect our decision [doctors], but it does affect the patient's […] The vast majority of patients ask for a CS because of the pain" (Hospital 4, OB/GYN).

When asked if the lack of epidural analgesia is a determinant of CS use, a midwife in a hospital with no access to an epidural and a high proportion of adolescents eloquently stated:

"Totally. I agree. We do not have a population of women aged 30 or more. They are empowered, and they can say: 'Yes, I would prefer a vaginal birth even though it means I will have pain during birth'. [Our population] are younger girls, and to be honest, a fifteen-year-old girl… it is tough to accept labour pain. We do not have access to an epidural for them to at least try a vaginal birth. In those cases, yes, we would recommend a CS" (Hospital 3, Midwife).

Others disagree: *"I don't think pain is per se a limitation during birth. We do have some strategies for pain management, especially midwives do. They know how to apply and use them. Thankfully, women can choose their companion during birth, and that is a big relief"* (Hospital 2 , OB/GYN).

In a hospital with access to an epidural, the key informants did not see birth pain as a CS determinant: *"We did not notice that pain [affects decision-making]. We do try to use epidurals, especially with younger girls […] But, no, not at all, I don't think pain influences CS"* (Hospital 1, Midwife).

Fear of litigation was relevant for the two professional groups with more experience– OB/GYN and midwives performing more than ten deliveries per week, Group 1 (N=151, 73%) and Group 2 (N=67, 65%) OB/GYNs and midwives currently in teaching, management, or research tasks (results not shown) while 40% and 37% of overall online survey responses disagreed and agreed, respectively (table 3).

"Unfortunately, as obstetricians, we are the most litigated specialists in the country. It's complex" (Hospital 2, OB/GYN).

"We have learnt that we are alone. Professional liability insurance does not cover us [in the case of litigation]. We must respond with our savings. Maybe with all your assets. We have learned that the lawyers defend the hospital in public hospitals, but not us, the professionals working in the hospital. You would think that they would defend the doctors, but they don't […]" (Hospital 1, Specialist).

There was consensus among all professional groups that the use of CS in public maternity hospitals is unrelated to financial incentives for the institution (N=621, 92%), the perception that CS is safer than vaginal birth (N=549, 81%), or unsupportive infrastructure (N=419, 74%) (Table 3).

Most of the participants did not recognise any formal communication of CS rates by their institutions. About half of the participants (N=380, 56·2%) expressed having regular meetings to audit clinical indications of CS. A quarter indicated that audit activities were in response to an unexpected adverse event rather than routine (N=161, 23%). Finally, six out of ten healthcare professionals agreed that there was a need to optimise CS use and half indicated they were familiar with the Robson Classification (data not shown).

For peer review only - http://bmjopen.bmj.com/site/about/guidelines.xhtml

Table 3 Online survey answers according to levels of agreement (N=655)

		Disagree or strongly disagree N (%)	Agree or strongly agree* N (%)	Neutral N (%)
	In my institution, the use of CS is associated with…			
Statements with higher % of high and moderate agreement	the complexity of our case load	138 (20·4)	411(60·8)	112 (16·5)
	financial incentives	621 (91·8)	14(2)	24 (3·5)
	CS being safer than a VB	549 (81·22)	40 (5·9)	71 (10·5)
	Shortage of human resources	522 (77·2)	86(12·7)	54 (7·9)
	deficits in infrastructure	419 (73·8)	89(13·1)	71 (10·5)
	inadequate number of trained healthcare professionals with the skills to perform complex deliveries	464 (68·6)	87(12·8)	98 (14·5)
	deficit in training to conduct VB after a CS	467 (69·0)	84 (12·4)	106 (15·6)
	lack of access to pain management strategies during VB**	163 (44)	116 (31)	85(12·7)
Statements with higher % of neutral	deficits in the training of residents to monitor and perform complex deliveries	418 (61·8)	104(15·3)	138 (20)
	fear of litigation	239 (39·1)	225(36·8)	147 (24)
	first time mothers and their anxiety and poor preparation for VB	246 (37·4)	221(33·7)	188 (28)
	women´s preferences	290 (44·2)	172(26·4)	193 (29·3)

*High and moderate agreement: more than 60% agree or disagree

** This statement was incorporated in the second round of surveys.

VB: vaginal birth; CS: caesarean section

Table 4 Online survey responses with the lowest level of agreement in clusters grouped by profession, years of experience, and clinical tasks (1)

	Cluster 1 Group 1 OB/GYN Specialists 11 years of experience N= 247	Group 2 OB/GYNs and midwives 22 years of experience antenatal care, research, and supervisory tasks N=103	Group 3 Residents N=167	Group 4 Midwifes N=138
**"I agree or completely agree that the use of CS is associated with deficits in the training of residents to monitor and perform complex deliveries"**	40 (16)	18(17)	8 (5)	39 (30)

For peer review only

**"I agree or completely agree that the use of CS in first time mothers is related to their anxiety as they don't know birth can be a long process"**	94(38)	30 (28)	57(34)	40(29)
**"I agree or completely agree that the use of CS is associated with women's preferences"**	74 (30)	26 (27)	48 (28·6)	24 (17)

(1) Cluster analysis Gower distance matrix partition around medoids (PAM)
CS: caesarean section; OB/GYN: Specialist in Obstetrics and Gynaecology

For peer review only

Discussion

This formative research study characterises public maternity hospitals' work environment in Argentina, emphasising the availability of essential obstetric services and human resources. It provides an improved understanding of healthcare providers' perceptions of determinants of CS in the public health system and a framework for anticipating which non-clinical interventions are feasible and valid considering the factors mentioned above.

Our research suggests that services are organized to provide adequate responses to obstetric emergencies. However, they informed deficits in the availability of strategies for pain management such as epidural, hot showers or relaxation techniques, and limited antenatal education. Some hospitals had insufficient human resources and space limitations impeding constant support during birth.

All the professional groups agreed that CS is related to complex-case mix or clinical indications. This finding is in line with international studies. Panda et al. summarised in a systematic review of 34 studies published in English the clinicians' views of factors influencing decision-making, and clinical indication appeared significant in eighteen studies. [8] Our online survey did not explore what providers understood as a clinical indication, but the semi-structured interviews further confirmed clinicians' views that increased comorbidities and new technologies contribute to the increased use of CS. These findings suggest that overall, healthcare teams perceived clinical indications as the main driver for performing a CS though about a third identified other contextual factors as determinants of CS use like services organisation, service provision, limited human resources, midwives' role in the group, women's preferences, adequate number of professionals to support women during labour, or deficits in antenatal education for both pregnant women and companions.

From the healthcare teams' perspective, the implementation of interventions targeted at clinicians, like audit and feedback, clinical guidelines, and the introduction of a second opinion may help to improve their clinical decision-making.[12–14] Still, this study showed the importance of assessing contextual factors such as services provision and human resources. If clinicians have limited options for pain

management or no human resources to provide continuous labour support –in some institutions without midwives–, the interventions focusing on clinical guidelines or audit and feedback alone may not lead to a desirable result.

Residents' skills emerged as a controversial theme in our study. Most residents perceived that their training to perform difficult vaginal births is not related to the increased use of CS, but more than half of midwives disagreed. As reported in many other countries, our data suggest that the use of instrumental deliveries is in decline, with consequently reduced possibilities for training new obstetricians.[28] How to better equip trainees for instrumental birth requires further research.[29]

We find differences in health providers' views of the role of women's preferences for CS. Midwives actively involved in antenatal care or assisting deliveries disagreed with the view that women's preferences are linked to CS use, which is also in line with previous research worldwide.[7–30] However, in half of the participating hospitals, midwives are not present to assist during birth. The power imbalance between medics and midwives is well documented. It can be addressed by changes in human resource organisation or by providing midwives with an active role on the team.[31,32] Our study did not explore power imbalance, but it showed that obstetricians lead labour in most hospitals, which results in a very limited role for midwives during birth, even in low-risk pregnancies. Once again, our study showed that the small number of midwives in most of the hospitals is insufficient to guarantee continuous professional support during birth for women. Considering the limited exposure to antenatal education in addition to this, the result is an environment which does not seem to facilitate the physiological process of vaginal birth.

The interviews with key informants acknowledged restrictive access to pain relief and its impact on the birth experience, particularly in adolescents. The management of pain during birth is an essential component of obstetric services and needs to be organised, resourced, and monitored. This is particularly true in Argentina, which has a 13% overall adolescent pregnancy rate and provinces with rates above 20%.[2] As well, a recurring topic that appeared when conducting semi-structured interviews was the need to improve antenatal education, which has already been identified as a non-clinical intervention with a positive impact on birth experience and vaginal birth rates.[33]

In terms of insufficient infrastructure or trained human resources, we mistakenly assumed that providers would acknowledge these factors as contributing to the overuse of CS. However, only providers in hospitals with severe shortage of human resources recognised the influence on clinical decision making.

The extent to which fear of litigation impacted obstetricians with more clinical experience requires all stakeholders to find possible solutions. There are extensive references to lawsuits against obstetricians in the literature and evidence .[8,9 34–36] We look at this potential determinant of CS as a burning issue in public maternity hospitals in Argentina. The fear of litigation was deeply rooted amongst experienced obstetricians in public hospitals as they feel they work in an environment with minimum support. The

For peer review only - http://bmjopen.bmj.com/site/about/guidelines.xhtml

road to sort out litigation in healthcare is complex compared to the experience of high-income countries but needs to be addressed if CS use is to be reduced in a sustainable manner.[37] The disagreement regarding the association between financial incentives and surgical deliveries is not surprising. This association is relevant for physicians paid in a fee-for-practice model.[7 38–40] In the public sector of Argentina, health care providers receive a fixed monthly salary regardless of the workload or the procedures they perform, and hospitals do not usually receive extra funding for a surgical delivery.

Strengths and limitations

To our knowledge, our study is the first to explore the perceptions of providers working in public hospitals in Argentina on the determinants of increasing trends of CS and future interventions to optimize its use. We showed that the use of formative research is a valuable tool to inform the design and implementation of future interventions. We had a large sample and representation of all professionals and obstetric tasks, which permitted us to identify differences across professional cadres. This variability allows for tailoring implementations specifically to each cadre, which will also benefit the implementation process. Participants' response rate was high considering the usual response rates from providers. However, professionals who did not complete the survey may not engage with the implementation process and therefore result in an additional barrier.

Another limitation is that opinions do not necessarily reflect current behaviour. Nevertheless, perceptions are an essential component of how health teams frame their environment and give sense to their clinical decisions.

Conclusions

There is a consensus among obstetricians, midwives, and residents on the need to implement interventions to decrease unnecessary surgical deliveries in Argentina.

We highlight an essential finding: obstetric services need to incorporate a holistic approach to pain management during vaginal birth and ensure continuous support during delivery from staff or carers to optimise CS use, especially in adolescents and first-time mothers.

At the same time, providers agreed that clinical indication is the main factor driving CS use and, therefore, interventions supporting the decision-making process can be of benefit.

Fear of litigation emerged as a critical issue highlighted by more experienced obstetricians. Therefore, strategies to protect health providers adhering to obstetric evidence-based guidelines against any legal matter within the public health system are warranted.

Author's contributions

All authors participated in developing this project protocol. SR was responsible for the scientific aspects of the project, coordinating the team, protocol development, as well as for writing and reviewing the final version of the original protocol. APB was responsible for the revision of the

For peer review only - http://bmjopen.bmj.com/site/about/guidelines.xhtml

protocol and for technical assistance regarding the design and methodology. She also obtained the funding for the project. MR, CP and YS were responsible for assisting the writing of the protocol and developing the versions of the instruments. NR and CG contributed to the development of the instruments and coordinated field work. CP, MR and YS contributed to the writing of the article and reviewed the final version. All authors provided feedback and revised the manuscript. The following authors were responsible for specific components of the project: SR wrote the first version of the study protocol and, together with MR, CP, and YS, coordinated its development and approved the final version. CP contributed to the design of the methodology and analysis of the project. MR contributed to the methodology, the design of the fieldwork. YS contributed to literature revision. YS, CS, CG and NR conducted the semi-structured interviews. CS and MR conducted the semi-structured interviews analysis. All authors read and approved the final manuscript.

Declaration of interest

The authors declare that there are no conflicts of interest.

Data sharing

The data is stored on CEDES' server, encrypted. CEDES is the guardian of the dataset. Data is anonymised. Our data collection forms did not include any variable that could reveal the identity of the participants. The datasets generated and/or analysed during the current study are available from the corresponding author on reasonable demand.

Acknowledgements

Source of funding

This research was funded by the UNDP-UNFPA-UNICEF-WHO-World Bank Special Programme of Research, Development and Research Training in Human Reproduction (HRP), a cosponsored programme executed by the World Health Organization (WHO) in the Department of Sexual and Reproductive Health and Research (SRH). Grant Number A65919

Dr Carla Perrotta also received funds to complete this research from the University College Dublin (UCD), College of Health Sciences, SEED Fund (2019) Grant number SF1735.

Natalia Righetti is a research fellow funded by the Ministry of Health, Buenos Aires City Government.

The study design; the collection, analysis, and interpretation of the data; the writing of the report and the decision to submit the paper for publication are solely the responsibility of the authors and do not reflect the views of the referred program or the other funding institutions. All authors had full access to all the data and accept responsibility to submit for publication.

Acknowledgements

Dr Marcelo Soria, Associate Professor, University of Buenos Aires provided valuable statistical advice.

Mercedes Vila Ortiz edited the manuscript.

For peer review only - http://bmjopen.bmj.com/site/about/guidelines.xhtml

References

1. Betrán AP, Ye J, Moller A-B, Zhang J, Gülmezoglu AM, Torloni MR. The Increasing Trend in Caesarean Section Rates: Global, Regional and National Estimates: 1990-2014. Zeeb H, editor. PLOS ONE. 2016 Feb 5;11(2):e M0148343.

2. Ministerio de Salud de la Nación Argentina. Deis | Dirección de Estadísticas e Información de Salud [Internet]. [accesed 2021 Jan 1]. Available from: http://www.deis.msal.gov.ar/

3. Rudey EL, Leal M do C, Rego G. Cesarean section rates in Brazil: Trend analysis using the Robson classification system. Medicine (Baltimore). 2020 Apr;99(17):e19880.

4. Carlson NS, Corwin EJ, Hernandez TL, Holt E, Lowe NK, Hurt KJ. Association between provider type and cesarean birth in healthy nulliparous laboring women: A retrospective cohort study. Birth Berkeley Calif. 2018 Jun;45(2):159–68.

5. Huang K, Tao F, Faragher B, Raven J, Tolhurst R, Tang S, et al. A mixed-method study of factors associated with differences in caesarean section rates at community level: The case of rural China. Midwifery. 2013 Aug;29(8):911–20.

6. Hoxha I, Syrogiannouli L, Braha M, Goodman DC, Da Costa BR, Jüni P. Caesarean sections and private insurance: Systematic review and meta-analysis. Vol. 7, BMJ Open. BMJ Publishing Group; 2017. Available from: https://pubmed-ncbi-nlm-nih-gov.ucd.idm.oclc.org/28827257/

7. Long Q, Kingdon C, Yang F, Renecle MD, Jahanfar S, Bohren MA, et al. Prevalence of and reasons for women's, family members', and health professionals' preferences for cesarean section in China: A mixed-methods systematic review. PLoS Med. 2018 Oct 1;15(10).

8. Panda S, Begley C, Daly D. Clinicians' views of factors influencing decision-making for caesarean section: A systematic review and metasynthesis of qualitative, quantitative and mixed methods studies. PLoS ONE. 2018 Jul 1;13(7):e0200941.

9. Panda S, Daly D, Begley C, Karlström A, Larsson B, Bäck L, et al. Factors influencing decision-making for caesarean section in Sweden - A qualitative study. BMC Pregnancy Childbirth. 2018 Sep 17;18(1).

10. Freitas PF, Fernandes TMB. Association between institutional factors, birth care profile, and cesarean section rates in Santa Catarina. Rev Bras Epidemiol Braz J Epidemiol. 2016 Sep;19(3):525–38.

11. Hodnett ED, Gates S, Hofmeyr GJ, Sakala C. Continuous support for women during childbirth. Cochrane Database Syst Rev. 2013 Jul 15;7:CD003766.

12. Chen I, Opiyo N, Tavender E, Mortazhejri S, Rader T, Petkovic J, et al. Non-clinical interventions for reducing unnecessary caesarean section. Vol. 2018, Cochrane Database of Systematic Reviews. John Wiley and Sons Ltd; 2018. p. CD005528.

13. Betran AP, Torloni MR, Zhang JJ, Gülmezoglu AM, WHO Working Group on Caesarean Section. WHO Statement on Caesarean Section Rates. BJOG Int J Obstet Gynaecol. 2016 Apr;123(5):667–70.

For peer review only

For peer review only - http://bmjopen.bmj.com/site/about/guidelines.xhtml

14. Opiyo N, Kingdon C, Oladapo OT, Souza JP, Vogel JP, Bonet M, et al. Non-clinical interventions to reduce unnecessary caesarean sections: WHO recommendations. Bull World Health Organ. 2020 Jan 1;98(1):66–8.

15. Ramos S, Romero M, Perrotta C, Sguassero Y, Straw C, Gialdini C, et al. Optimizing the use of cesarean section in Argentina: design and methodology of a formative research for the development of interventions. Reprod Health. 2021 Dec 26;18(1):23.

16. Leirbakk MJ, Torper J, Engebretsen E, Opsahl JN, Zeanah P, Magnus JH. Formative research in the development of a salutogenic early intervention home visiting program integrated in public child health service in a multiethnic population in Norway 11 Medical and Health Sciences 1117 Public Health and Health Services. BMC Health Serv Res. 2018 Sep 27;18(1).

17. Zimmerman M, Steckel L. Formative research: pretesting, revising, and more pretesting. Dev Commun Rep. 1985;(51):9, 12.

18. Vastine A, Gittelsohn J, Ethelbah B, Anliker J, Caballero B. Formative research and stakeholder participation in intervention development. Am J Health Behav. 2005;29(1):57–69.

19. Karolinski A, Mercer R, Salgado P, Ocampo C, Bolzán A, Nieto R, et al. Primer informe nacional de relevamiento epidemiológico SIP-Gestión: desarrollo e implementación a escala nacional de un sistema de información en salud de la mujer y perinatal en Argentina. OPS; 2013.

20. Karolinski A, Mercer R, Bolzán A, Salgado P, Ocampo C, Nieto R, et al. Foundations for the development and implementation of a women's and perinatal health information model for management in Latin America. Rev Panam Salud PublicaPan Am J Public Health. 2018;42.

21. Hollin IL, Craig BM, Coast J, Beusterien K, Vass C, DiSantostefano R, et al. Reporting Formative Qualitative Research to Support the Development of Quantitative Preference Study Protocols and Corresponding Survey Instruments: Guidelines for Authors and Reviewers. The Patient. 2020 Feb;13(1):121–36.

22. O'Brien BC, Harris IB, Beckman TJ, Reed DA, Cook DA. Standards for Reporting Qualitative Research. Acad Med. 2014 Sep;89(9):1245–51.

23. Robson M, Hartigan L, Murphy M. Methods of achieving and maintaining an appropriate caesarean section rate. Best Pract Res Clin Obstet Gynaecol. 2013 Apr;27(2):297–308.

24. Van Der Maaten L, Hinton G. Visualizing Data using t-SNE. Vol. 9, Journal of Machine Learning Research. 2008.

25. Reynolds AP, Richards G, De La Iglesia B, Rayward-Smith VJ. Clustering rules: A comparison of partitioning and hierarchical clustering algorithms. J Math Model Algorithms. 2006 Dec 3;5(4):475–504.

26. R Core Team (2019). — European Environment Agency [Internet]. [cited 2020 Jul 3]. Available from: https://www.eea.europa.eu/data-and-maps/indicators/oxygen-consuming-substances-in-rivers/r-development-core-team-2006

27. Gale NK, Heath G, Cameron E, Rashid S, Redwood S. Using the framework method for the analysis of qualitative data in multi-disciplinary health research. BMC Med Res Methodol. 2013 Dec 18;13(1):117.

For peer review only - http://bmjopen.bmj.com/site/about/guidelines.xhtml

28. Kyser KL, Lu X, Santillan D, Santillan M, Caughey AB, Wilson MC, et al. Forceps Delivery Volumes in Teaching and Nonteaching Hospitals: Are Volumes Sufficient for Physicians to Acquire and Maintain Competence? Acad Med J Assoc Am Med Coll. 2014 Jan;89(1):71–6.

29. Rose K, Kwan L, Pluym ID, Zhang H, Han CS, Afshar Y. Forceps-assisted vaginal delivery: the landscape of obstetrics and gynecology resident training. J Matern-Fetal Neonatal Med Off J Eur Assoc Perinat Med Fed Asia Ocean Perinat Soc Int Soc Perinat Obstet. 2019 Oct 21;1–7.

30. Loke AY, Davies L, Mak YW. Is it the decision of women to choose a cesarean section as the mode of birth? A review of literature on the views of stakeholders. BMC Pregnancy Childbirth. 2019 Aug;19(1).

31. Kingdon C, Downe S, Betran AP. Interventions targeted at health professionals to reduce unnecessary caesarean sections: a qualitative evidence synthesis. BMJ Open. 2018 Dec 16;8(12):e025073.

32. Kingdon C, Downe S, Betran AP. Non-clinical interventions to reduce unnecessary caesarean section targeted at organisations, facilities and systems: Systematic review of qualitative studies. 2018; Available from: https://doi.org/10.1371/journal.pone.0203274

33. Kingdon C, Downe S, Betran AP. Women's and communities' views of targeted educational interventions to reduce unnecessary caesarean section: A qualitative evidence synthesis. Vol. 15, Reproductive Health. BioMed Central Ltd.; 2018. Available from: https://pubmed.ncbi.nlm.nih.gov/30041661/

34. Johanson R, Newburn M, Macfarlane A. Has the medicalisation of childbirth gone too far? Vol. 324, British Medical Journal. BMJ Publishing Group; 2002. p. 892–5.

35. Carroll AE, Buddenbaum JL. High and low-risk specialties experience with the U.S. medical malpractice system. BMC Health Serv Res. 2013;13(1):465.

36. Durrance CP, Hankins S. Medical Malpractice Liability Exposure and OB/GYN Physician Delivery Decisions. Health Serv Res. 2018 Aug 1;53(4):2633–50.

37. Cardoso R, Zarin W, Nincic V, Barber SL, Gulmezoglu AM, Wilson C, et al. Evaluative reports on medical malpractice policies in obstetrics: a rapid scoping review. Syst Rev. 2017 Dec 6;6(1):181.

38. Bogg L, Huang K, Long Q, Shen Y, Hemminki E. Dramatic increase of Cesarean deliveries in the midst of health reforms in rural China. Soc Sci Med. 2010 May;70(10):1544–9.

39. Tadevosyan M, Ghazaryan A, Harutyunyan A, Petrosyan V, Atherly A, Hekimian K. Factors contributing to rapidly increasing rates of cesarean section in Armenia: A partially mixed concurrent quantitative-qualitative equal status study. BMC Pregnancy Childbirth. 2019 Jan 3;19(1):2.

40. Melman S, Schreurs RHP, Dirksen CD, Kwee A, Nijhuis JG, Smeets NAC, et al. Identification of barriers and facilitators for optimal cesarean section care: perspective of professionals. BMC Pregnancy Childbirth. 2017 Dec 14;17(1):230.

For peer review only

For peer review only - http://bmjopen.bmj.com/site/about/guidelines.xhtml

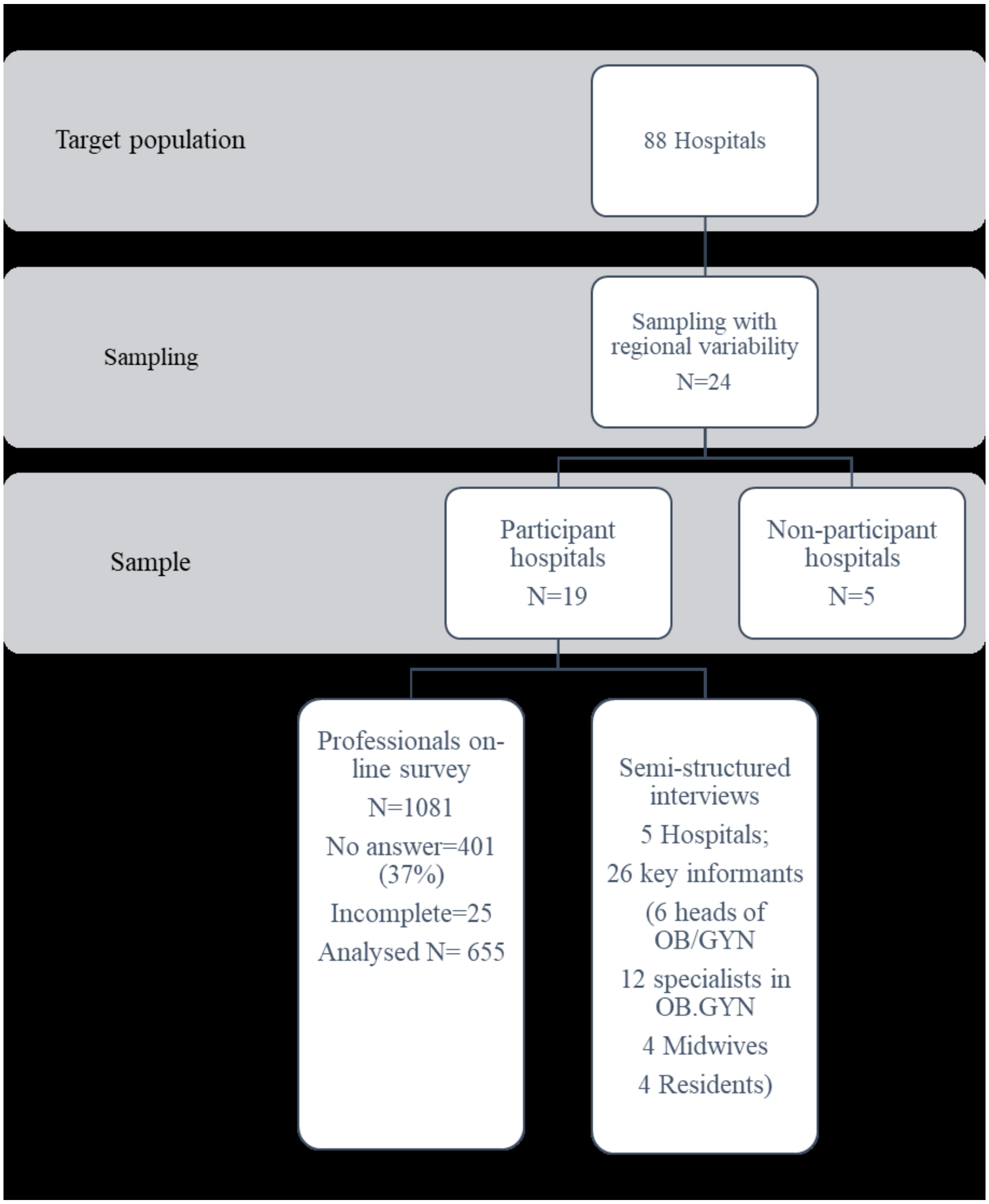

Figure 1 Study flowchart

BMJ Open

Caesarean birth in public maternities in Argentina: a formative research study on the views of obstetricians, midwives, and trainees.

Journal:	*BMJ Open*
Manuscript ID	bmjopen-2021-053419.R1
Article Type:	Original research
Date Submitted by the Author:	24-Nov-2021
Complete List of Authors:	Perrotta, Carla; University College Dublin, School of Public Health, Physiotherapy and Sport Sciences<br>Romero, Mariana; CONICET (Consejo Nacional de Investigaciones Científicas y Tecnológicas)<br>Sguassero, Yanina; Centro Rosarino de Estudios Perinatales<br>Straw, Cecilia; CEDES, School of Social Sciences, University of Buenos Aires<br>Gialdini, Celina; Centro Rosarino de Estudios Perinatales<br>Righetti, Natalia; CEDES, Health, Economy and Society<br>Betran, Ana Pilar; World Health Organization, Department of Reproductive Health and Research<br>Ramos, Silvina; CEDES, Health, Economy and Society
**Primary Subject Heading**:	Obstetrics and gynaecology
Secondary Subject Heading:	Health services research
Keywords:	Maternal medicine < OBSTETRICS, PUBLIC HEALTH, Organisation of health services < HEALTH SERVICES ADMINISTRATION & MANAGEMENT

SCHOLARONE™
Manuscripts

I, the Submitting Author has the right to grant and does grant on behalf of all authors of the Work (as defined in the below author licence), an exclusive licence and/or a non-exclusive licence for contributions from authors who are: i) UK Crown employees; ii) where BMJ has agreed a CC-BY licence shall apply, and/or iii) in accordance with the terms applicable for US Federal Government officers or employees acting as part of their official duties; on a worldwide, perpetual, irrevocable, royalty-free basis to BMJ Publishing Group Ltd ("BMJ") its licensees and where the relevant Journal is co-owned by BMJ to the co-owners of the Journal, to publish the Work in this journal and any other BMJ products and to exploit all rights, as set out in our licence.

The Submitting Author accepts and understands that any supply made under these terms is made by BMJ to the Submitting Author unless you are acting as an employee on behalf of your employer or a postgraduate student of an affiliated institution which is paying any applicable article publishing charge ("APC") for Open Access articles. Where the Submitting Author wishes to make the Work available on an Open Access basis (and intends to pay the relevant APC), the terms of reuse of such Open Access shall be governed by a Creative Commons licence – details of these licences and which Creative Commons licence will apply to this Work are set out in our licence referred to above.

Other than as permitted in any relevant BMJ Author's Self Archiving Policies, I confirm this Work has not been accepted for publication elsewhere, is not being considered for publication elsewhere and does not duplicate material already published. I confirm all authors consent to publication of this Work and authorise the granting of this licence.

For peer review only

Caesarean birth in public maternities in Argentina: a formative research study on the views of obstetricians, midwives, and trainees.

Perrotta Carla, Romero Mariana, Sguassero Yanina, Straw Cecilia, Gialdini Celina, Righetti Natalia, Betrán Ana-Pilar, Ramos Silvina

Carla Perrotta, PhD. Corresponding author. School of Public Health, University College Dublin, National University of Ireland, Dublin, Ireland. Address: Woodview House, Belfield campus. E-mail: carla.perrotta@ucd.ie +353 83 84 99147

Mariana Romero, MD. Centro de Estudios de Estado y Sociedad (CEDES)- CONICET (Consejo Nacional de Investigaciones Científicas y Tecnológicas), Buenos Aires, Argentina. E-mail: mromero@cedes.org

Yanina Sguassero, PhD. Centro Rosarino de Estudios Perinatales, Rosario, Argentina. E-mail: ysguassero@crep.org.ar

Cecilia Straw, PhD. Facultad de Ciencias Sociales, Universidad de Buenos Aires. Centro de Estudios de Estado y Sociedad (CEDES)-, Buenos Aires, Argentina. E-mail: Cecilia.straw@gmail.com

Celina Gialdini, MD. Centro Rosarino de Estudios Perinatales, Rosario, Argentina. E-mail: cgialdini@crep.org.ar.

Natalia Righetti, MSc. Centro de Estudios de Estado y Sociedad (CEDES), Buenos Aires, Argentina. E-mail: natirighetti@gmail.com

Ana Pilar Betrán, MD. UNDP, UNFPA, UNICEF, WHO, World Bank Special Program of Research, Development and Research Training in Human Reproduction, Department of Sexual and Reproductive Health and Research (HRP), World Health Organization, Geneva, Switzerland. E-mail: betrana@who.int

Silvina Ramos, MSc. Centro de Estudios de Estado y Sociedad (CEDES), Buenos Aires, Argentina. E-mail: silvinaramosarcoiris@gmail.com

For peer review only

Abstract

Objectives To explore obstetricians', midwives', and trainees' perceptions of CS determinants in the context of public obstetric care services provision in Argentina. Our hypothesis is that known determinants of CS use may differ in settings with limited access to essential obstetric services.

Setting We conducted a formative research study in nineteen public maternity hospitals in Argentina. An institutional survey assessed the availability of essential obstetric services. Subsequently, we conducted online surveys and semi-structured interviews to assess the opinions of providers on known CS determinants.

Results

Obstetric services showed an adequate provision of emergency obstetric care but limited services to support women during birth. Midwives, with some exceptions, are not involved during labour. We received 680 surveys from obstetricians, residents, and midwives (response rate of 63%) and interviewed 26 key informants. Six out of ten providers (411, 61%) indicated that the use of CS is associated with *the complexities of our caseload*. Limited pain management access was deemed a potential contributing factor for CS in adolescents and first-time mothers. Providers have conflicting views on the adequacy of training to deal with complex or prolonged labour. Obstetricians with more than ten years of clinical experience indicated that fear of litigation was also associated with CS. Overall, there is consensus on the need to implement interventions to reduce unnecessary CS.

Conclusions

Public Maternity Hospitals in Argentina have made significant improvements in the provision of emergency services. The environment of service provision does not seem to facilitate the physiological process of vaginal birth. Providers acknowledged some of these challenges.

Funding

UNDP, UNFPA, UNICEF, WHO, World Bank Special Program of Research, Development and Research Training in Human Reproduction, Department of Sexual and Reproductive Health and Research (HRP), World Health Organization. University College Dublin Seed Funding.

Trial registration IS002316

For peer review only

Article Summary

Strengths and limitations of this study

- This study is one of the few studies exploring the perceptions of providers working in low-resourced settings in middle-income countries on the determinants of increasing trends of CS.
- Large sample and representation of all professionals and obstetric tasks working in low-resourced settings.
- Cluster analysis allowed researchers to describe the response variability across professional groups in relation to specific CS determinants.
- The use of Formative Research is a valuable tool to inform the design and implementation of future interventions.
- Even though the response rate was good (63%), those who did not respond may have different views on the determinants of CS.

For peer review only

Background

In recent decades, there has been a sustained and unprecedented increase in caesarean section (CS) rates worldwide, in particular −but not exclusively− in the Latin America region (1). Argentina, a middle-income country, reported rates between 27% and 52% within the public sector in 2017, while official data from the private sector is unavailable (2). According to reports by the Perinatal Reporting System, from 2009 to 2017, the use of CS has increased in the public sector by 22%, from 28% to 34%, with striking rates in some provinces being close to 50% (2). Similarly, Brazil registered national rates of 55%, with private providers close to 90% (3).

The determination of the ideal CS rate remains controversial. A WHO systematic review of ecological studies concluded that CS rates above the threshold of 9-16% were not associated with decreases in maternal and infant mortality (4). Another global analysis led by the WHO also concluded that once the CS rate reaches 10%, further increases had no impact on maternal, neonatal, and infant mortality rates. Although the exact ideal CS rate is unknown, the WHO states that at population level, CS rates higher than 10% are not associated with reductions in maternal and newborn mortality rates - thus, current regional trends in Latin America cause concern and call for a scaling-up of interventions to reduce unnecessary CS (5,6).

There are multiple factors underpinning the increase in CS rates (7). Notably, financial incentives, the organisation of obstetric services, obstetricians' fear of litigation, and professional attitudes towards CS (8–13). Systematic reviews show that, contrary to perceived opinion, women prefer to give birth vaginally (14,15). According to a recent scoping review, women's preferences for CS are in the range of 5% to 20% depending on the female population characteristics, i.e. age, ethnicity, cultural

For peer review only - http://bmjopen.bmj.com/site/about/guidelines.xhtml

background, social and economic status, level of education, and at what stage in the pregnancy that question is asked or how the question of preference is framed (15).

Along with clinical interventions, non-clinical interventions such as antenatal education, continuous emotional support during labour, audit and feedback, and the implementation of clinical guidelines have shown modest but statistically significant reductions in CS rates (7,16,17). A quality improvement study conducted in Brazil presented encouraging results. The project demonstrated a reduction in CS rates through a multilevel intervention targeting healthcare professionals. The interventions included reinforcing the use of analgesia during birth, continuous professional education and feedback over CS rates to the teams, among other measures (18). The World Health Organization (WHO) also recommends implementing non-clinical interventions to reduce unnecessary surgical births and it encourages the incorporation of an initial formative research process to tailor interventions to local contexts, ensure implementation feasibility, and anticipate specific barriers or identify facilitators (19,20).

Argentina is a federal country with a two-tier health system: a public system financed by government funds and a private system mix of social insurance and private insurance. Virtually all births occur at healthcare institutions (99.3%), and 64% of them within the public health system. Specialists lead obstetric services in Obstetrics and Gynaecology (OB/GYN) while the role of midwives depends on how the hospital organises its services and human resources. Midwives provide antenatal care and depending on the institution, can assist or lead low-risk vaginal births.

We conducted formative research to enrich understanding of the reasons for the sustained increase in CS use in Argentina's public sector with the aim to subsequently use this knowledge to tailor interventions in Argentina (21). We applied a mixed-methods approach to explore multiple layers and perspectives of both women and health providers, and to assess the characteristics of the obstetric services in the participating hospitals including the availability of and access to pain management interventions (e.g. anaesthesia and non-pharmacological interventions), and the availability of and access to emergency obstetric services. Formative research has its origins in ethnographic studies and uses various data-gathering processes. The rationale behind this approach is to consider the peculiarities of the setting to better tailor suitable interventions and, at the same time, to catalyse the target population into change (22–24).

This article will focus on the perspectives of specialists in OB/GYN, midwives and OB/GYN residents (trainees) on determinants of CS use in Argentina's public maternity hospitals.

Methods

For peer review only - http://bmjopen.bmj.com/site/about/guidelines.xhtml

A comprehensive description of the methodology has been published elsewhere (21). Briefly, we used a research approach that combined quantitative and qualitative data gathering techniques conducted by a multidisciplinary team.

The contextual factors of the study refer to the adequacy of services to provide emergency obstetric care, human resources availability, and women's access to comprehensive obstetric care like antenatal education, companionship during birth, and holistic pain management.

The eligibility criteria for hospitals to participate in the study were: a) public hospitals with ≥1000 annual childbirths, and b) use of the National Perinatal Information System (25,26). Out of the 88 potentially eligible institutions, 24 were non-randomly selected to represent the six Argentinian regions. Nineteen agreed to participate and obtained approval from local Ethics Committees (Figure 1), and five institutions declined to participate. Finally, five Argentinian regions were included in this study: Centre, North, Northeast and Buenos Aires City. In 2016, the median CS rate in the 24 eligible hospitals was 37%, ranging between 27% and 52% (2).

The project was approved by the Independent Ethics Committee of Centro Rosarino de Estudios Perinatales and by the provincial Ethics Committees and/or the Teaching and Research Committees at each of the selected hospitals pursuant to the requirements in each jurisdiction. It was also approved by the Research Project Review Panel of the UNDP/UNFPA/UNICEF/WHO/World Bank Special Programme of Research, Development and Research Training in Human Reproduction (HRP) at the Department of Sexual and Reproductive Health and Research of WHO, and the WHO Research Ethics Review Committee, Geneva, Switzerland. Reference Number A65919. In Argentina, the research protocol was registered in the RENIS database (number IS002316).

The report of this research follows the Standards for Reporting Qualitative Research and Formative Research and the STROBE cross sectional checklist (27–29).

For peer review only

Subjects and data collection

Multiple data sources were used: an institutional survey, a health care provider online survey, and semi-structured interviews with key informants. The head of OB/GYN identified a spokesperson who led the fieldwork and introduced the project to health team members at each participating hospital.

Institutional survey

The selected champion in each hospital completed the institutional survey. The survey gathered information on the number of live births in 2017, the number and type of providers involved in maternal care, hospital CS rates, institutional infrastructures such as the number of beds, availability of services to safely manage obstetric emergencies, availability of antenatal education for women, and pain management during labour and birth.

For peer review only - http://bmjopen.bmj.com/site/about/guidelines.xhtml

Online survey for healthcare providers

Eligible participants for the online survey included all specialised physicians, obstetricians, and residents at Obstetrics & Gynaecology services at participating hospitals. Both permanent staff and shift personnel were included.

The health professionals' online survey included participation consent (mandatory answer) and 32 questions: 26 with pre-established options and six open-ended questions. The survey gathered the following data: role and healthcare work, including the number of deliveries/caesarean sections performed in a week; perception of determining factors for the use of caesarean section related to the institution, health professionals and pregnant women; frequency of use of monitoring cycles; improvement in quality of care; and Robson Classification knowledge, among others (30).

The piloting process involved ten hospitals and asked participants to indicate if they would like to add anything. Fifteen providers suggested that inadequate pain management during labour is a contributing factor; thus, we incorporated it in the list of determinants of CS use ("*lack of pain management interventions is associated with the use of CS*").

We sent an email with the link to the survey to each eligible subject who provided an email address; the text included a brief presentation of the study with a consent form. The respondents' gender or sex was not recorded to ensure anonymity. There were three weekly reminders. Fieldwork was carried out from July 2018 to August 2019.

Semi-structured interviews for key informants

Out of the 19 hospitals participating in the study, we drew a convenience sample of five hospitals representing the different regions. In those hospitals, we conducted in-depth interviews after the on-line survey with key informants, including the heads of departments or services, specialists in OB/GYN, midwives, and residents. The interviewers were researchers with experience in qualitative methods and in maternal health and organisational culture. The interviews were conducted either in person or by video call and recorded under informed consent.

Data analysis

We described variables using standard statistical parameters. Continuous variables were presented as means and standard deviations (SDs), whereas categorical variables were presented as numbers and percentages. Human resources data was standardised to 1000 live births in 2017.

For the online survey, answers were grouped into three categories: disagreement (strongly disagree and disagree), neutral, and agreement (completely agree and agree), and were described using frequencies. The statements with modest consensus –those with ≤ 50% agreement or disagreement– were analysed using cluster analysis to examine if professional groups or any institutional variable explained the variance. Given the different types of variables assessed, a Gower's distance matrix was calculated. It

For peer review only - http://bmjopen.bmj.com/site/about/guidelines.xhtml

was visualised using the t-Distributed Stochastic Neighbour Embedding (t-SNE, 1) and clustered with Partition around medoids (PAM) with k = 4 (31,32). All statistical analyses were performed using R version 3.6.3 (33).

We transcribed the recorded in-depth interviews. Transcripts were independently coded by two researchers with experience in qualitative analysis. We undertook an in-depth framework analysis of the qualitative data focusing on determinants of CS and institutional factors (34). We compared key themes emerging from the interviews with the online survey results, expanding to supplement survey findings when appropriate. Throughout the results section, we report verbatim extracts to validate and illustrate specific issues, provide further explanations, or include themes that were not addressed by the online survey.

Patient and public involvement

The development of the protocol used evidence from qualitative research exploring women's experiences with obstetric services in public maternity hospitals. Once the study has been published, the results will be disseminated for professional, non-professional audiences in participant provinces, as well as to non-governmental organizations overseeing maternal care in the Latin American region.

Results

Women giving birth in the 19 participant hospitals represented 19% of the total live births in Argentina's public sector in 2017 (Figure 1). The hospitals corresponded to the North region (four hospitals), the Northeast (two hospitals), the centre (seven hospitals), and capital city (six hospitals). Five hospitals (three from the West-Cuyo Region and two from the South) either declined to participate or did not complete the endorsement by their ethics committee.

Table 1 shows the characteristics of the 19 hospitals in 2017 as reported by the key informants in each institution. The percentage of live births by CS in the study hospitals ranged from 29% to 40% (Table 1). The hospitals had similar services; all of them had laboratory, neonatal intensive care units, adequate access to emergency CS, and resources to manage obstetric emergencies, including safe blood transfusion services. All hospitals but one had limited access to pain management during birth, insufficient provision of antenatal education, and restricted companionship during CS. They differed on the number of healthcare providers per 1,000 live births, with a median of seven obstetricians/1000 live births and 2·8 midwives/1000 live births. Two hospitals reported a very low staffing ratio −one hospital reported 1 obstetrician/1000 live births−; and the largest number of obstetricians was 23/1000 live births in one hospital. All the professionals received a fixed monthly salary unrelated to performance.

For peer review only

For peer review only - http://bmjopen.bmj.com/site/about/guidelines.xhtml

Table 1 Characteristics of participant hospitals (N=19)

Variables	Hospitals<br>N (%) Mean (SD) Median (range)
Type of institution	
Maternity	7 (37%)
General hospital	12 (63%)
Number of beds †	
Obstetrics	44 (1-93)
Neonatal Intensive Care Unit	18 (1-50)
Number of delivery rooms or units of labour, delivery, and recovery†	6 (1-16)
Number of live births	52633<br>2355 (1025-9633) †
Caesarean Sections (CS)	
Number of CS	21102
CS rate†	37.2 (27-50)
Protocol for vaginal delivery after CS	15 (83%)
Residency Program	
OB/GYNs	18 (90%)
Midwives	7 (36%)
Number of professionals†	
OB/GYNs	20 (2-67)
Residents†	13 (0-31)
Midwives†	12 (0-30)
Number of professionals/ 1000 live births†	
OB/GYNs	7 (1-23)
Residents	5.8 (0-15)
Midwives	2.8 (0-18)
Midwives' availability (24/7)	13 (68%)
Midwives participating in low-risk births	9 (45%)
Pain management interventions	
Access to non-opioids	10 (52%)
Access to epidural (24/7)	9 (47.4%)
Access to opioids	12 (63%)
Relaxation techniques	4 (21.1%)
Access to hot shower	9 (47.4%)
Access to massages	3 (15.2%)
Access to companionship (24/7)	
During labour	14 (73%)
During delivery	16 (84%)
During CS	2 (10%)
Availability of prenatal education (morning, afternoon, Monday through Friday)	1 (5.5%)

*Data is shown as N (%) unless otherwise indicated.
†median (range)
OB/GYN: Specialist in Obstetrics and Gynaecology

We received 680 online surveys (response rate of 63%; range 19%-84%). We excluded 25 due to missing data related to professional background, which resulted in 655 completed surveys to analyse (Figure 1).

The completed surveys were from OB/GYN specialists (N=327), midwives (N=161) and residents (N=167). Cluster analysis allowed us to identify four groups based on tasks and years of experience, and profession. Group 1 (N=247) included OB/GYNs who worked for a mean of 11 years and attended 2-10 births per week (any type). Group 2 had more experienced OB/GYNs (N=80) and midwives (N=23) (mean 22 years of professional experience) involved either in antenatal care, teaching, or management duties. Group 3 consisted of residents (N=167) with three years of experience participating in more than ten deliveries per week. Group 4 included midwives (N=138) with a mean professional

For peer review only - http://bmjopen.bmj.com/site/about/guidelines.xhtml
For peer review only

practice of 12·5 years in assisting vaginal deliveries (Table 2). We conducted 26 semi-structured interviews in total with six heads of OB/GYN departments, 12 specialists in OB/GYN, four midwives and four residents representing each of the regions in this sample (North -2 hospitals-, East, Centre and Buenos Aires City).

Table 2 Online survey clusters according to clinical experience (years) and main duties

	Median years of professional experience	Main duties	N=655
**Group 1 OB/GYNs**	11	Assistance of all delivery modes. 2-10 deliveries per week	247
**Group 2 OB/GYNs and midwifes**	22	Antenatal care, research, supervisory tasks, education	103
**Group 3 Residents**	3·5	Assistance of all delivery modes. More than 10 deliveries per week	167
**Group 4 Midwifes**	12·5	Assistance of all deliveries 2-10 deliveries per week	138

OB/GYN: Specialist in Obstetrics and Gynaecology

Six out of ten participants (411, 61%) agreed that the use of CS is associated with "*the complexities of our caseload*" (Table 3). Additionally, key informants point out diagnostic innovations and comorbidities as determinants of CS use.

"Nowadays, we have more diagnostic procedures during pregnancy […] Maybe, in the past, women had a vaginal birth with worse clinical outcomes […] yes, the number of CS has increased, but the neonatal outcomes have improved" (Hospital 2, OB/GYN).

"The main factor for CS is clinical. We see more women with severe comorbidities. Also, if they have had a previous CS and comorbidity, the delivery is a CS […]." (Hospital 3, OB/GYN).

Shortage of skilled professionals was considered a determinant of the overuse of CS by 12.8% of the sample, while 16% agreed that insufficient training for complicated births was a determinant.

A similar level of agreement appeared regarding inadequate skills to conduct vaginal birth after CS (VBAC) (N=84, 12.4 %) (Table 3). However, when asked if CS is associated with residents' lack of training in performing complex deliveries, the professional groups differed in their answers: 30% of midwives agreed that there is an association (N=39, 30%) while only 5% of residents agreed (Table 4).

The semi-structured interviews allowed us to explore further whether the number, skills, and experience of obstetricians, midwives, and residents influence the use of CS. Among the interviewees, there was consensus that professionals are well trained; however, midwives perceived themselves as less prone to intervene and considered that the medical professionals were trained with a more interventionist mindset.

For peer review only - http://bmjopen.bmj.com/site/about/guidelines.xhtml

For peer review only

"We have to fight every day as doctors tend to intervene. Now we have a training program on foetal medicine, and most doctors perform a foetal ultrasound. I notice more and more the tendency to intervene" (Hospital 1, midwife).

The semi-structured interviews also revealed challenges in training future generations of obstetricians to perform instrumental deliveries when needed.

"Instrumental deliveries are tough, and no one wants to do them. Before, it was a common practice; now, people are cautious. In the collective imagination of society, the forceps can kill or harm the baby. Even judges, lawyers have that idea… [And the training to conduct instrumental deliveries?] … little as we don't do them" (Hospital 4, OB/GYN).

"A resident can complete their training -if lucky- performing five forceps. Maybe a handful of obstetricians at the ward have the skills to use forceps. Doctors prefer a CS as it can quickly solve the clinical problem" (Hospital 2, Resident).

In most hospitals, key informants did not support the idea that scarcity of human resources is relevant to the increase of CS. However, in two hospitals with either no midwives on their teams or a very low staff-to-live-birth ratio, clinicians did recognise it as a contributing factor. In the latter two hospitals, busy shifts with only one doctor on-call were described. In those circumstances, if a woman presented with failing-to-progress labour, the decision may switch to a surgical delivery (verbatim not shown).

Questions related to women's preferences and first-time mothers' anxiety due to lack of preparation for birth had the highest number of neutral responses. We found variability between professional groups. 17% of midwives agreed with the statement "*women demand a CS"* compared to 30% of obstetricians and 28% of residents. For the statement "*the use of CS in first-time mothers is related to anxiety*", 38% of the obstetricians, 34.4% of residents and 29% of midwives agreed it could be considered a determinant of CS use (Table 4).

Key informants discussed both matters:

"*We have requests both ways. Patients that want vaginal birth at all cost and patients that want CS. We work under significant pressure. Patients have changed…*" (Hospital 4, OB/GYN).

Lack of adequate access to pain management interventions as a reason for CS did not reach consensus in the online survey; 31% agreed, 44% disagreed, and 12.7% had a neutral opinion, with statistically insignificant variation between responses (Table 3). Key informants helped to understand the role of limited access to epidurals and other pain management strategies like hot water, relaxation, or massage. There was some consensus that the absence of these options might influence women's requests for a CS, especially in adolescent mothers.

"If a patient is in pain, I won't specifically say 'OK, this patient needs a CS because of the pain'. It does not affect our decision [doctors], but it does affect the patient's […] The vast majority of patients ask for a CS because of the pain" (Hospital 4, OB/GYN).

When asked if the lack of epidural analgesia is a determinant of CS use, a midwife in a hospital with no access to an epidural and a high proportion of adolescents eloquently stated:

For peer review only - http://bmjopen.bmj.com/site/about/guidelines.xhtml

"Totally. I agree. We do not have a population of women aged 30 or more. They are empowered, and they can say: 'Yes, I would prefer a vaginal birth even though it means I will have pain during birth'. [Our population] are younger girls, and to be honest, a fifteen-year-old girl… it is tough to accept labour pain. We do not have access to an epidural for them to at least try a vaginal birth. In those cases, yes, we would recommend a CS" (Hospital 3, Midwife).

Others disagree: *"I don't think pain is per se a limitation during birth. We do have some strategies for pain management, especially midwives do. They know how to apply and use them. Thankfully, women can choose their companion during birth, and that is a big relief"* (Hospital 2, OB/GYN).

In a hospital with access to an epidural, the key informants did not see birth pain as a CS determinant: *"We did not notice that pain [affects decision-making]. We do try to use epidurals, especially with younger girls […] But, no, not at all, I don't think pain influences CS"* (Hospital 1, Midwife).

Fear of litigation was relevant for the two professional groups with more experience– OB/GYN and midwives performing more than ten deliveries per week, Group 1 (N=151, 73%), and OB/GYNs and midwives currently in teaching, management, or research tasks, Group 2 (N=67, 65%) (results not shown)— while 40% and 37% of overall online survey responses disagreed and agreed, respectively (Table 3).

"Unfortunately, as obstetricians, we are the most litigated specialists in the country. It's complex" (Hospital 2, OB/GYN).

"We have learnt that we are alone. Professional liability insurance does not cover us [in the case of litigation]. We must respond with our savings. Maybe with all your assets. We have learned that the lawyers defend the hospital in public hospitals, but not us, the professionals working in the hospital. You would think that they would defend the doctors, but they don't […]" (Hospital 1, Specialist).

There was consensus among all professional groups that the use of CS in public maternity hospitals is unrelated to financial incentives for the institution (N=621, 92%), the perception that CS is safer than vaginal birth (N=549, 81%), or unsupportive infrastructure (N=419, 74%) (Table 3).

Most of the participants did not recognise any formal communication of CS rates by their institutions. About half of the participants (N=380, 56·2%) expressed having regular meetings to audit clinical indications of CS. A quarter indicated that audit activities were in response to an unexpected adverse event rather than routine (N=161, 23%). Finally, six out of ten healthcare professionals agreed that there was a need to optimise CS use and half indicated they were familiar with the Robson Classification with no differences in terms of their opinion on the influence of clinical caseload and the knowledge of the Robson classification (data not shown).

For peer review only - http://bmjopen.bmj.com/site/about/guidelines.xhtml

Table 3 Online survey answers according to levels of agreement (N=655)

		Disagree or strongly disagree N (%)	Agree or strongly agree* N (%)	Neutral N (%)
	In my institution, the use of CS is associated with…			
Statements with higher % of high and moderate agreement	the complexity of our case load	138 (20.4)	411(60.8)	112 (16.5)
	financial incentives	621 (91.8)	14(2)	24 (3.5)
	CS being safer than a VB	549 (81.2)	40 (5.9)	71 (10.5)
	Shortage of human resources	522 (77.2)	86(12.7)	54 (7.9)
	deficits in infrastructure	419 (73.8)	89(13.1)	71 (10.5)
	inadequate number of trained healthcare professionals with the skills to perform complex deliveries	464 (68.6)	87(12.8)	98 (14.5)
	deficit in training to conduct VB after a CS	467 (69)	84 (12.4)	106 (15.6)
	lack of access to pain management strategies during VB**	163 (44)	116 (31)	85(12.7)
Statements with higher % of neutral answers or low level of	deficits in the training of residents to monitor and perform complex deliveries	418 (61.8)	104(15.3)	138 (20)
	fear of litigation	239 (39.1)	225(36.8)	147 (24)
	first time mothers and their anxiety and poor preparation for VB	246 (37.4)	221(33.7)	188 (28)
	women´s preferences	290 (44.2)	172(26.4)	193 (29.3)

*High and moderate agreement: more than 60% agree or disagree
** This statement was incorporated in the second round of surveys.
VB: vaginal birth; CS: caesarean section

Table 4 Online survey responses with the lowest level of agreement in clusters grouped by profession, years of experience, and clinical tasks (1)

	Cluster 1 Group 1 OB/GYN Specialists 11 years of experience N= 247	Group 2 OB/GYNs and midwives 22 years of experience antenatal care, research, and supervisory tasks N=103	Group 3 Residents N=167	Group 4 Midwifes N=138
"I agree or completely agree that the use of CS is associated with deficits in the training of residents to monitor and perform complex deliveries"	40 (16)	18(17)	8 (5)	39 (30)
"I agree or completely agree that the use of CS in first time mothers is related to their anxiety as they don't know birth can be a long process"	94(38)	30 (28)	57(34)	40(29)
"I agree or completely agree that the use of CS is associated with women's preferences"	74 (30)	26 (27)	48 (28.6)	24 (17)

(1) Cluster analysis Gower distance matrix partition around medoids (PAM)
CS: caesarean section; OB/GYN: Specialist in Obstetrics and Gynaecology

Discussion

This formative research study characterises public maternity hospitals' work environment in Argentina, emphasising the availability of essential obstetric services and human resources. It provides an improved understanding of healthcare providers' perceptions of determinants of CS in the public health system and a framework for anticipating which non-clinical interventions are feasible and valid considering the factors mentioned above.

Our research suggests that services are organized to provide adequate responses to obstetric emergencies. However, they informed deficits in the availability of strategies for pain management such as epidural, hot showers or relaxation techniques, and limited antenatal education, all of which are important issues for women (35). Some hospitals had insufficient human resources and space limitations impeding constant support during birth. Evidence worldwide suggests fear of pain or lack of pain relief during labour as one of the primary factors underlying women preference for CS (7,15,36,37). Our formative research further reinforces the importance for women to have access to pain management strategies. The findings indicate that women from these hospitals prefer vaginal birth over CS, but CS preference is influenced by the pain experienced during birth (38).

For peer review only - http://bmjopen.bmj.com/site/about/guidelines.xhtml

The interviews with key informants acknowledged restrictive access to pain relief and its impact on the birth experience, particularly in adolescents. The management of pain during birth is an essential component of obstetric services and needs to be organised, resourced, and monitored. This is particularly true in Argentina, which has a 13% overall adolescent pregnancy rate and provinces with rates above 20% (2). As well, a recurring topic that appeared when conducting semi-structured interviews was the need to improve antenatal education, which has already been identified as a non-clinical intervention with a positive impact on birth experience and vaginal birth rates (7,35,39).

All the professional groups agreed that CS is related to complex-case mix or clinical indications. This finding is in line with results from other international studies (11). Panda et al. summarised clinicians' views of factors influencing decision-making in a systematic review of 34 studies published in English, and clinical indication appeared significant in eighteen studies (11). Still, the reported CS rates by most of the hospitals in this sample - median of 37% - suggest that case-mix alone cannot explain the high rates. Our online survey did not explore what providers understood as a clinical indication, but the semi-structured interviews further confirmed clinicians' views that increased comorbidities and new technologies contribute to the increased use of CS. These findings suggest that overall, healthcare teams perceived clinical indications as the main driver for performing a CS though about a third identified other contextual factors as determinants of CS use like services organisation, service provision, limited human resources, midwives' role, women's preferences, adequate number of professionals to support women during labour, or deficits in antenatal education for both pregnant women and companions. Though factors such an increase in the age of women, in the proportion of nulliparous women, in the prevalence of obesity, or in multiple births (*in vitro fertilization)* have been suggested as culprits of the increasing trend of the use of CS, these factors alone cannot explain the phenomenon. The WHO emphasizes that non-clinical factors such as convenience of a CS, fear of litigation and other organizational issues need to be recognized and addressed for successful interventions (39).

In addition, the WHO recommends the use of the Robson classification to assess, monitor and compare CS rates in a standardized and action-oriented manner (19,30). This classification helps to understand the obstetric case mixed of a maternity and thus can challenge some common myths about causes of increasing CS rate such as obstetrical condition conducive to CS. In our survey, only about half of the healthcare providers had knowledge of the Robson classification. Interventions to increase knowledge and use of the Robson classification could be beneficial in these maternities. Since our study was not designed to analyse how the knowledge of the Robson classification may influence clinicians' opinion on the use of CS or the current rates, it is not possible to make inferences on the association between these two variables.

From the healthcare teams' perspective, the implementation of interventions targeted at clinicians like audit and feedback, clinical guidelines, and the introduction of a second opinion may help to improve their clinical decision-making. Still, this study showed the importance of assessing contextual factors

For peer review only - http://bmjopen.bmj.com/site/about/guidelines.xhtml

such as services provision and human resources. If clinicians have limited options for pain management or no human resources to provide continuous labour support –in some institutions without midwives–, the interventions focusing on clinical guidelines or audit and feedback alone may not lead to a desirable result.

Residents' skills emerged as a controversial theme in our study. Most residents perceived that their training to perform difficult vaginal births is not related to the increased use of CS, but more than half of midwives disagreed. As reported in many other countries, our data suggest that the use of instrumental deliveries is in decline, with consequently reduced possibilities for training new obstetricians. How to better equip trainees for instrumental birth requires further research (40,41).

We find differences in health providers' views of the role of women's preferences for CS. Midwives actively involved in antenatal care or assisting deliveries disagreed with the view that women's preferences are linked to CS use, which is also in line with previous research worldwide. However, in half of the participating hospitals, midwives are not present to assist during birth. The power imbalance between medics and midwives is well documented. It can be addressed by changes in human resource organisation or by providing midwives with an active role on the team. Our study did not explore power imbalance, but it showed that obstetricians lead labour in most hospitals, which results in a very limited role for midwives during birth, even in low-risk pregnancies (8,16). Once again, our study showed that the small number of midwives in most of the hospitals is insufficient to guarantee continuous professional support during birth for women. Considering the limited exposure to antenatal education in addition to this, the result is an environment which does not seem to facilitate the physiological process of vaginal birth.

In terms of insufficient infrastructure or trained human resources, we mistakenly assumed that providers would acknowledge these factors as contributing to the overuse of CS. However, only providers in hospitals with severe shortage of human resources recognised the influence on clinical decision making. The extent to which fear of litigation impacted obstetricians with more clinical experience requires all stakeholders to find possible solutions. There are extensive references to lawsuits against obstetricians in the literature and evidence. We look at this potential determinant of CS as a burning issue in public maternity hospitals in Argentina. The fear of litigation was deeply rooted amongst experienced obstetricians in public hospitals as they feel they work in an environment with minimum support. The road to sort out litigation in healthcare is complex compared to the experience of high-income countries but needs to be addressed if CS use is to be reduced in a sustainable manner (42–44).The disagreement regarding the association between financial incentives and surgical deliveries is not surprising. This association is relevant for physicians paid in a fee-for-practice model. In the public sector of Argentina, health care providers receive a fixed monthly salary regardless of the workload or the procedures they perform, and hospitals do not usually receive extra funding for a surgical delivery.

For peer review only - http://bmjopen.bmj.com/site/about/guidelines.xhtml

Strengths and limitations

To our knowledge, our study is the first to explore the perceptions of providers working in public hospitals in Argentina on the determinants of increasing trends of CS and future interventions to optimize its use. We showed that the use of formative research is a valuable tool to inform the design and implementation of future interventions. We had a large sample and representation of all professionals and obstetric tasks, which permitted us to identify differences across professional cadres. This variability allows for tailoring implementations specifically to each cadre, which will also benefit the implementation process. Participants' response rate was high considering the usual response rates from providers. However, professionals who did not complete the survey may not engage with the implementation process and therefore result in an additional barrier.

Another limitation is that opinions do not necessarily reflect current behaviour. Nevertheless, perceptions are an essential component of how health teams frame their environment and give sense to their clinical decisions.

Conclusions

There is a consensus among obstetricians, midwives, and residents on the need to implement interventions to decrease unnecessary surgical deliveries in Argentina.

We highlight an essential finding: obstetric services need to incorporate a holistic approach to pain management during vaginal birth and ensure continuous support during delivery from staff or carers to optimise CS use, especially in adolescents and first-time mothers.

At the same time, providers agreed that clinical indication is the main factor driving CS use and, therefore, interventions supporting the decision-making process can be of benefit.

Fear of litigation emerged as a critical issue highlighted by more experienced obstetricians. Therefore, strategies to protect health providers adhering to obstetric evidence-based guidelines against any legal matter within the public health system are warranted.

Figure 1 Flowchart of the Hospital selection for the on-line survey component and semi-structured interviews

Author's contributions

All authors participated in developing this project protocol. SR was responsible for the scientific aspects of the project, coordinating the team, protocol development, as well as for writing and reviewing the final version of the original protocol. APB was responsible for the revision of the protocol and for technical assistance regarding the design and methodology. She also obtained the funding for the project. MR, CP and YS were responsible for assisting the writing of the protocol and developing the versions of the instruments. NR and CG contributed to the development of the instruments and

For peer review only - http://bmjopen.bmj.com/site/about/guidelines.xhtml

coordinated field work. CP, MR and YS contributed to the writing of the article and reviewed the final version. All authors provided feedback and revised the manuscript. The following authors were responsible for specific components of the project: SR wrote the first version of the study protocol and, together with MR, CP, and YS, coordinated its development and approved the final version. CP contributed to the design of the methodology and analysis of the project. MR contributed to the methodology, the design of the fieldwork. YS contributed to literature revision. YS, CS, CG and NR conducted the semi-structured interviews. CS and MR conducted the semi-structured interviews analysis. All authors read and approved the final manuscript.

Declaration of interest

The authors declare that there are no conflicts of interest.

Data sharing

The data is stored on CEDES' server, encrypted. CEDES is the guardian of the dataset. Data is anonymised. Our data collection forms did not include any variable that could reveal the identity of the participants. The datasets generated and/or analysed during the current study are available from the corresponding author on reasonable demand.

Acknowledgements

Source of funding

This research was funded by the UNDP-UNFPA-UNICEF-WHO-World Bank Special Programme of Research, Development and Research Training in Human Reproduction (HRP), a cosponsored programme executed by the World Health Organization (WHO) in the Department of Sexual and Reproductive Health and Research (SRH). WHO Project Number A65919

Dr Carla Perrotta also received funds to complete this research from the University College Dublin (UCD), College of Health Sciences, SEED Fund (2019) Grant number SF1735.

Natalia Righetti is a research fellow funded by the Ministry of Health, Buenos Aires City Government. The study design; the collection, analysis, and interpretation of the data; the writing of the report and the decision to submit the paper for publication are solely the responsibility of the authors and do not reflect the views of the referred program or the other funding institutions. All authors had full access to all the data and accept responsibility to submit for publication.

Acknowledgements

Dr Marcelo Soria, Associate Professor, University of Buenos Aires provided valuable statistical advice.

Mercedes Vila Ortiz edited the manuscript.

Carolyn Ingram edited the revised version of the manuscript.

For peer review only

Ethics statement

The project was approved by the Independent Ethics Committee of Centro Rosarino de Estudios Perinatales and by the provincial Ethics Committees and/or the Teaching and Research Committees at each of the selected hospitals pursuant to the requirements in each jurisdiction. It was also approved by the Research Project Review Panel of the UNDP/UNFPA/UNICEF/WHO/World Bank Special Programme of Research, Development and Research Training in Human Reproduction (HRP) at the Department of Sexual and Reproductive Health and Research of WHO, and the WHO Research Ethics Review Committee, Geneva, Switzerland. Reference Number A65919. In Argentina, the research protocol was registered in the RENIS database (number IS002316).

References

1. Betran AP, Ye J, Moller A-B, Souza JP, Zhang J. Trends and projections of caesarean section rates: global and regional estimates. BMJ Glob Health. 2021 Jun 1;6(6):e005671.

2. Ministerio de Salud de la Nación Argentina. Dirección de Estadísticas e Información de la Salud [Internet]. [cited 2021 Nov 20]. Available from: https://www.argentina.gob.ar/salud/deis [accessed 2021 Nov 20].

3. Rudey EL, Leal M do C, Rego G. Cesarean section rates in Brazil: Trend analysis using the Robson classification system. Medicine (Baltimore). 2020 Apr;99(17):e19880.

4. Betran AP, Torloni MR, Zhang J, Ye J, Mikolajczyk R, Deneux-Tharaux C, et al. What is the optimal rate of caesarean section at population level? A systematic review of ecologic studies. Reprod Health. 2015 Jun 21;12:57.

5. Ye J, Zhang J, Mikolajczyk R, Torloni MR, Gülmezoglu AM, Betran AP. Association between rates of caesarean section and maternal and neonatal mortality in the 21st century: a worldwide population-based ecological study with longitudinal data. BJOG Int J Obstet Gynaecol. 2016 Apr;123(5):745–53.

6. Ye J, Betrán AP, Guerrero Vela M, Souza JP, Zhang J. Searching for the optimal rate of medically necessary cesarean delivery. Birth Berkeley Calif. 2014 Sep;41(3):237–44.

7. Betrán AP, Temmerman M, Kingdon C, Mohiddin A, Opiyo N, Torloni MR, et al. Interventions to reduce unnecessary caesarean sections in healthy women and babies. Lancet Lond Engl. 2018 Oct 13;392(10155):1358–68.

8. Carlson NS, Corwin EJ, Hernandez TL, Holt E, Lowe NK, Hurt KJ. Association between provider type and cesarean birth in healthy nulliparous laboring women: A retrospective cohort study. Birth Berkeley Calif. 2018 Jun;45(2):159–68.

9. Huang K, Tao F, Faragher B, Raven J, Tolhurst R, Tang S, et al. A mixed-method study of factors associated with differences in caesarean section rates at community level: the case of rural China. Midwifery. 2013 Aug 1;29(8):911–20.

For peer review only

10. Hoxha I, Braha M, Syrogiannouli L, Goodman DC, Jüni P. Caesarean section in uninsured women in the USA: systematic review and meta-analysis. BMJ Open. 2019 Mar 3;9(3):e025356.

11. Panda S, Begley C, Daly D. Clinicians' views of factors influencing decision-making for caesarean section: A systematic review and metasynthesis of qualitative, quantitative and mixed methods studies. PLoS ONE. 2018 Jul;13(7):e0200941.

12. Freitas PF, Fernandes TMB. Association between institutional factors, birth care profile, and cesarean section rates in Santa Catarina. Rev Bras Epidemiol Braz J Epidemiol. 2016 Sep;19(3):525–38.

13. Long Q, Kingdon C, Yang F, Renecle MD, Jahanfar S, Bohren MA, et al. Prevalence of and reasons for women's, family members', and health professionals' preferences for cesarean section in China: A mixed-methods systematic review. PLOS Med. 2018 Oct 16;15(10):e1002672.

14. Mazzoni A, Althabe F, Gutierrez L, Gibbons L, Liu NH, Bonotti AM, et al. Women's preferences and mode of delivery in public and private hospitals: a prospective cohort study. BMC Pregnancy Childbirth. 2016 Feb 8;16(1):34.

15. Coates D, Thirukumar P, Spear V, Brown G, Henry A. What are women's mode of birth preferences and why? A systematic scoping review. Women Birth J Aust Coll Midwives. 2020 Jul;33(4):323–33.

16. Hodnett ED, Gates S, Hofmeyr GJ, Sakala C. Continuous support for women during childbirth. Cochrane Database Syst Rev. 2013 Jul 15;7:CD003766.

17. Chen I, Opiyo N, Tavender E, Mortazhejri S, Rader T, Petkovic J, et al. Non-clinical interventions for reducing unnecessary caesarean section. Cochrane Database Syst Rev. 2018 Sep 28;9:CD005528.

18. Negrini R, Ferreira RD da S, Albino RS, Daltro CAT. Reducing caesarean rates in a public maternity hospital by implementing a plan of action: a quality improvement report. BMJ Open Qual. 2020 May;9(2):e000791.

19. Betran AP, Torloni MR, Zhang JJ, Gülmezoglu AM, WHO Working Group on Caesarean Section. WHO Statement on Caesarean Section Rates. BJOG Int J Obstet Gynaecol. 2016 Apr;123(5):667–70.

20. World Health Organization. WHO statement on caesarean section rates [Internet]. [cited 2021 Nov 20]. Available from: http://www.who.int/reproductivehealth/publications/maternal_perinatal_health/cs-statement/en/ [accessed 2021 Nov 20]

21. Ramos S, Romero M, Perrotta C, Sguassero Y, Straw C, Gialdini C, et al. Optimizing the use of cesarean section in Argentina: design and methodology of a formative research for the development of interventions. Reprod Health. 2021 Jan 26;18(1):23.

22. Leirbakk MJ, Torper J, Engebretsen E, Opsahl JN, Zeanah P, Magnus JH. Formative research in the development of a salutogenic early intervention home visiting program integrated in public child health service in a multiethnic population in Norway. BMC Health Serv Res. 2018 Sep 27;18(1):741.

For peer review only - http://bmjopen.bmj.com/site/about/guidelines.xhtml

23. Zimmerman M, Steckel L. Formative research: pretesting, revising, and more pretesting. Dev Commun Rep. 1985;(51):9, 12.

24. Vastine A, Gittelsohn J, Ethelbah B, Anliker J, Caballero B. Formative research and stakeholder participation in intervention development. Am J Health Behav. 2005 Feb;29(1):57–69.

25. Karolinski A, Mercer R, Bolzan A, Salgado P, Ocampo C, Nieto R, et al. Foundations for the development and implementation of a women's and perinatal health information model for management in Latin America/ Fundamentos do desenvolvimento e implementacao de um modelo de informacao em saude da materna e perinatal orientado a gestao na America Latina. Rev Panam Salud Publica. 2018 Apr 1;42(1):e148.

26. Karolinski A, Mercer R, Micone P, Mazzoni A, Bolzan A, Wainer V. Importancia del uso de indicadores epidemiológicos para mejorar la calidad de atención en salud materna y perinatal en hospitales públicos de la Argentina: la experiencia de la Red Perinatal AMBA. Programa Actual En Ginecol Obstet B Aires Ed Panam. 2008;

27. Hollin IL, Craig BM, Coast J, Beusterien K, Vass C, DiSantostefano R, et al. Reporting Formative Qualitative Research to Support the Development of Quantitative Preference Study Protocols and Corresponding Survey Instruments: Guidelines for Authors and Reviewers. The Patient. 2020 Feb;13(1):121–36.

28. O'Brien BC, Harris IB, Beckman TJ, Reed DA, Cook DA. Standards for reporting qualitative research: a synthesis of recommendations. Acad Med J Assoc Am Med Coll. 2014 Sep;89(9):1245–51.

29. von Elm E, Altman DG, Egger M, Pocock SJ, Gøtzsche PC, Vandenbroucke JP, et al. Strengthening the Reporting of Observational Studies in Epidemiology (STROBE) statement: guidelines for reporting observational studies. BMJ. 2007 Oct 20;335(7624):806–8.

30. Robson M, Hartigan L, Murphy M. Methods of achieving and maintaining an appropriate caesarean section rate. Best Pract Res Clin Obstet Gynaecol. 2013 Apr;27(2):297–308.

31. Maaten L van der, Hinton G. Visualizing Data using t-SNE. J Mach Learn Res. 2008;9(86):2579–605.

32. Reynolds AP, Richards G, de la Iglesia B, Rayward-Smith VJ. Clustering Rules: A Comparison of Partitioning and Hierarchical Clustering Algorithms. J Math Model Algorithms. 2006 Dec 1;5(4):475–504.

33. R Core Team. R: A language and environment for statistical computing [Internet]. Vienna, Austria; 2020. Available from: https://www.R-project.org/

34. Gale NK, Heath G, Cameron E, Rashid S, Redwood S. Using the framework method for the analysis of qualitative data in multi-disciplinary health research. BMC Med Res Methodol. 2013 Sep 18;13(1):117.

35. Kingdon C, Downe S, Betran AP. Non-clinical interventions to reduce unnecessary caesarean section targeted at organisations, facilities and systems: Systematic review of qualitative studies. PLOS ONE. 2018 Sep 4;13(9):e0203274.

36. Stoll KH, Hauck YL, Downe S, Payne D, Hall WA, International Childbirth Attitudes- Prior to Pregnancy (ICAPP) Study Team. Preference for cesarean section in young nulligravid women in eight OECD countries and implications for reproductive health education. Reprod Health. 2017 Sep 12;14(1):116.

For peer review only - http://bmjopen.bmj.com/site/about/guidelines.xhtml

37. Colomar M, Opiyo N, Kingdon C, Long Q, Nion S, Bohren MA, et al. Do women prefer caesarean sections? A qualitative evidence synthesis of their views and experiences. PloS One. 2021;16(5):e0251072.

38. Perrotta C, Romero M, Sguassero Y, Riguetti N, Gialdini C, Betran AP, et al. Women's Views and Preferences for Mode of Birth in Public Hospitals in Argentina: a Mixed-methods Study. Research Square [Preprint]. 2021 [cited 2021 Nov 08]. Available from: https://www.researchsquare.com/article/rs-180797/v1

39. Opiyo N, Kingdon C, Oladapo OT, Souza JP, Vogel JP, Bonet M, et al. Non-clinical interventions to reduce unnecessary caesarean sections: WHO recommendations. Bull World Health Organ. 2020 Jan 1;98(1):66–8.

40. Skinner S, Davies-Tuck M, Wallace E, Hodges R. Perinatal and Maternal Outcomes After Training Residents in Forceps Before Vacuum Instrumental Birth. Obstet Gynecol. 2017 Jul;130(1):151–8.

41. Kyser KL, Lu X, Santillan D, Santillan M, Caughey AB, Wilson MC, et al. Forceps Delivery Volumes in Teaching and Nonteaching Hospitals: Are Volumes Sufficient for Physicians to Acquire and Maintain Competence? Acad Med J Assoc Am Med Coll. 2014 Jan;89(1):71–6.

42. Rudey EL, Leal M do C, Rego G. Defensive medicine and cesarean sections in Brazil. Medicine (Baltimore). 2021 Jan 8;100(1):e24176.

43. Minkoff H. Fear of litigation and cesarean section rates. Semin Perinatol. 2012 Oct;36(5):390–4.

44. Cardoso R, Zarin W, Nincic V, Barber SL, Gulmezoglu AM, Wilson C, et al. Evaluative reports on medical malpractice policies in obstetrics: a rapid scoping review. Syst Rev. 2017 Sep 6;6(1):181.

For peer review only

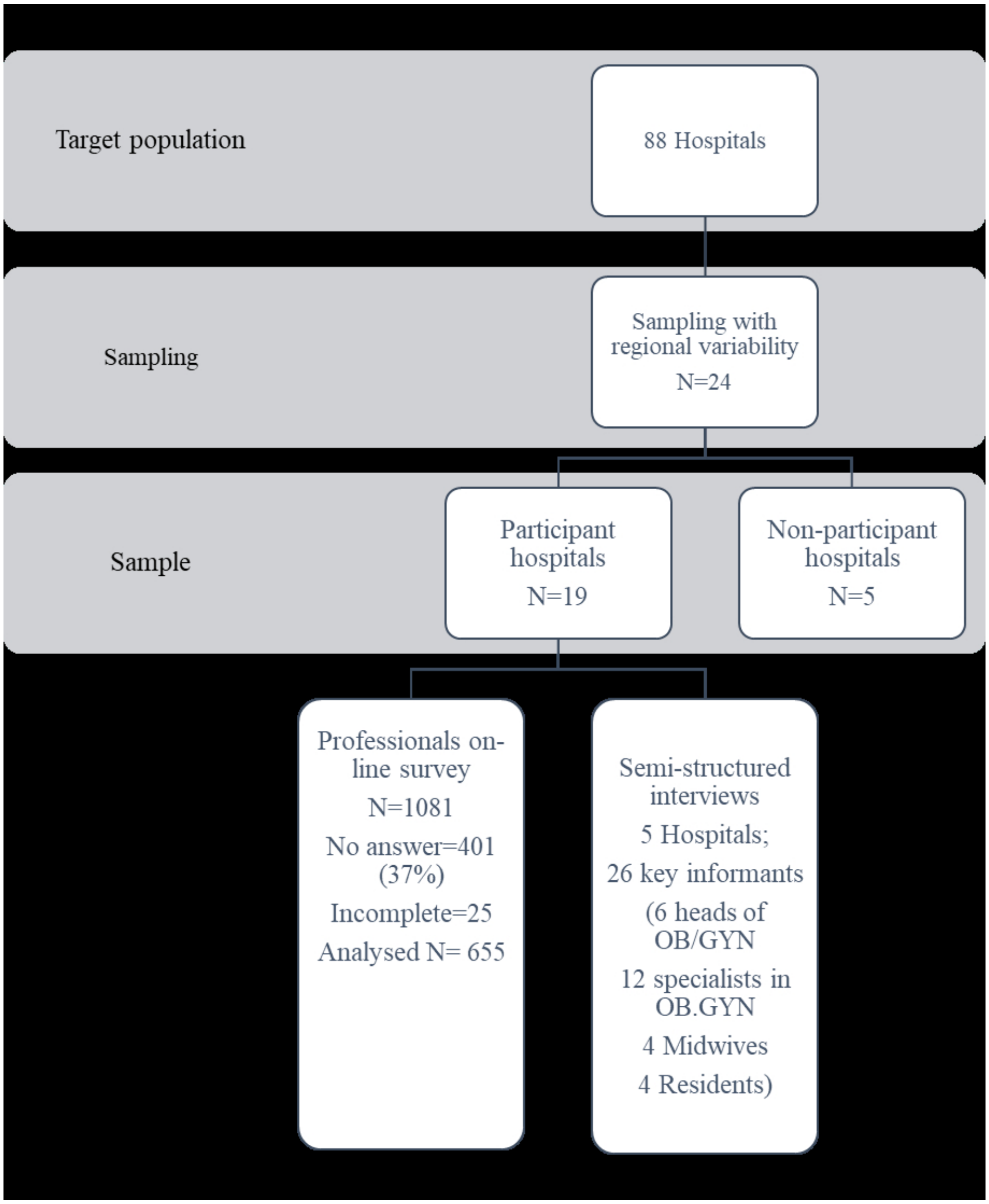

Figure 1 Study flowchart

STROBE Statement—checklist of items that should be included in reports of observational studies

	Item No	Recommendation	Page No
**Title and abstract**	1	(*a*) Indicate the study's design with a commonly used term in the title or the abstract	1
		(*b*) Provide in the abstract an informative and balanced summary of what was done and what was found	2
**Introduction**			
Background/rationale	2	Explain the scientific background and rationale for the investigation being reported	3
Objectives	3	State specific objectives, including any prespecified hypotheses	4
**Methods**			
Study design	4	Present key elements of study design early in the paper	3
Setting	5	Describe the setting, locations, and relevant dates, including periods of recruitment, exposure, follow-up, and data collection	5
Participant	6	(*Cross-sectional study*—Give the eligibility criteria, and the sources and methods of selection of participants	5
Variables	7	Clearly define all outcomes, exposures, predictors, potential confounders, and effect modifiers. Give diagnostic criteria, if applicable	na
Data sources/ measurement	8*	For each variable of interest, give sources of data and details of methods of assessment (measurement). Describe comparability of assessment methods if there is more than one group	*6*<br>*na*
Bias	9	Describe any efforts to address potential sources of bias	6
Study size	10	Explain how the study size was arrived at	
Quantitative variables	11	Explain how quantitative variables were handled in the analyses. If applicable, describe which groupings were chosen and why	
Statistical methods	12	(*a*) Describe all statistical methods, including those used to control for confounding	6
		(*b*) Describe any methods used to examine subgroups and interactions	
		(*c*) Explain how missing data were addressed	
		(*Cross-sectional study*—If applicable, describe analytical methods taking account of sampling strategy	
		(*e*) Describe any sensitivity analyses	

Continued on next page

For peer review only - http://bmjopen.bmj.com/site/about/guidelines.xhtml

**Results**			
Participants	13*	(a) Report numbers of individuals at each stage of study—eg numbers potentially eligible, examined for eligibility, confirmed eligible, included in the study, completing follow-up, and analysed	6
		(b) Give reasons for non-participation at each stage	
		(c) Consider use of a flow diagram	
Descriptive data	14*	(a) Give characteristics of study participants (eg demographic, clinical, social) and information on exposures and potential confounders	7
		(b) Indicate number of participants with missing data for each variable of interest	
		(c) *Cohort study*—Summarise follow-up time (eg, average and total amount)	
Outcome data	15*	*Cohort study*—Report numbers of outcome events or summary measures over time	*na*
		Case-control study—Report numbers in each exposure category, or summary measures of exposure	*na*
		Cross-sectional study—Report numbers of outcome events or summary measures	*7*
Main results	16	(*a*) Give unadjusted estimates and, if applicable, confounder-adjusted estimates and their precision (eg, 95% confidence interval). Make clear which confounders were adjusted for and why they were included	na
		(*b*) Report category boundaries when continuous variables were categorized	7-8
		(*c*) If relevant, consider translating estimates of relative risk into absolute risk for a meaningful time period	na
Other analyses	17	Report other analyses done—eg analyses of subgroups and interactions, and sensitivity analyses	
**Discussion**			
Key results	18	Summarise key results with reference to study objectives	9
Limitations	19	Discuss limitations of the study, taking into account sources of potential bias or imprecision. Discuss both direction and magnitude of any potential bias	10
Interpretation	20	Give a cautious overall interpretation of results considering objectives, limitations, multiplicity of analyses, results from similar studies, and other relevant evidence	10
Generalisability	21	Discuss the generalisability (external validity) of the study results	10
**Other information**			
Funding	22	Give the source of funding and the role of the funders for the present study and, if applicable, for the original study on which the present article is based	11

*Give information separately for cases and controls in case-control studies and, if applicable, for exposed and unexposed groups in cohort and cross-sectional studies.

Note: An Explanation and Elaboration article discusses each checklist item and gives methodological background and published examples of transparent reporting. The STROBE checklist is best used in conjunction with this article (freely available on the Web sites of PLoS Medicine at http://www.plosmedicine.org/, Annals of Internal Medicine at http://www.annals.org/, and Epidemiology at http://www.epidem.com/). Information on the STROBE Initiative is available at www.strobe-statement.org.

For peer review only

For peer review only - http://bmjopen.bmj.com/site/about/guidelines.xhtml
